# ADAR-mediated RNA editing of DNA:RNA hybrids is required for DNA double strand break repair

**DOI:** 10.1038/s41467-021-25790-2

**Published:** 2021-09-17

**Authors:** Sonia Jimeno, Rosario Prados-Carvajal, María Jesús Fernández-Ávila, Sonia Silva, Domenico Alessandro Silvestris, Martín Endara-Coll, Guillermo Rodríguez-Real, Judit Domingo-Prim, Fernando Mejías-Navarro, Amador Romero-Franco, Silvia Jimeno-González, Sonia Barroso, Valeriana Cesarini, Andrés Aguilera, Angela Gallo, Neus Visa, Pablo Huertas

**Affiliations:** 1 Departamento de Genética, Universidad de Sevilla, Sevilla, 41080 Spain; 2 Centro Andaluz de Biología Molecular y Medicina Regenerativa-CABIMER, Universidad de Sevilla-CSIC-Universidad Pablo de Olavide, Sevilla, 41092 Spain; 3 RNA Editing Lab, Oncohaematology Department, IRCCS Ospedale Pediatrico “Bambino Gesù”, Viale San Paolo 15, 00146 Rome, Italy; 4 Department of Molecular Biosciences, The Wenner-Gren Institute, Stockholm University, 10691 Stockholm, Sweden; 5 Present Address: Moirai Biodesign SL, Parc Científic de Barcelona, 08028 Barcelona, Spain

**Keywords:** Homologous recombination, RNA editing

## Abstract

The maintenance of genomic stability requires the coordination of multiple cellular tasks upon the appearance of DNA lesions. RNA editing, the post-transcriptional sequence alteration of RNA, has a profound effect on cell homeostasis, but its implication in the response to DNA damage was not previously explored. Here we show that, in response to DNA breaks, an overall change of the Adenosine-to-Inosine RNA editing is observed, a phenomenon we call the RNA Editing DAmage Response (REDAR). REDAR relies on the checkpoint kinase ATR and the recombination factor CtIP. Moreover, depletion of the RNA editing enzyme ADAR2 renders cells hypersensitive to genotoxic agents, increases genomic instability and hampers homologous recombination by impairing DNA resection. Such a role of ADAR2 in DNA repair goes beyond the recoding of specific transcripts, but depends on ADAR2 editing DNA:RNA hybrids to ease their dissolution.

## Abstract (web summary)

Different roles of specific RNA-related factors in DNA repair have now been reported. Here the authors reveal a role for RNA-editing by ADAR in DNA end resection following double strand break formation and a change in pattern of ADAR2-mediated A-to-I editing.

## Introduction

Cells are continuously challenged by DNA damage. Among all kinds of insults that a DNA molecule has to deal with, double-strand breaks (DSBs) are the most dangerous. Indeed, just one unrepaired DSB is enough to either kill or terminally arrest cells. For these reasons when DSBs are formed, a complex cellular response—the DNA damage response (DDR)—is triggered in order to ensure the proper repair of such a threat to genomic integrity^1^.

There are several pathways that can be used in order to repair a DSB and the choice between them is highly regulated. A eukaryotic cell can repair a DSB either by the simple re-ligation of the DNA ends (a process known as Non-Homologous End-Joining, NHEJ)^2^ or by a homology-driven repair event. There are different routes among the repair pathways that use homologous regions for repair, all of which are grouped in a process called homologous recombination (HR)^3^. All HR events share a first biochemical step called DNA resection, which is the key to decide the pathway that will be eventually used to repair the DSB^4,5^. This process consists of the nucleolytic degradation of the DNA ends of the break that produces tails of 3' ended single-stranded DNA (ssDNA), that are rapidly protected by the RPA protein complex.

In recent years, the importance of RNA and RNA-related factors in DNA repair has become clear^6–9^. Indeed, many RNA-related proteins have been shown to be targets of the DNA damage-induced post-translational modifications^10–12^. Also, direct roles of specific RNA-related factors in DNA repair have been recently reported (for a review see^9^). Moreover, the RNA molecule itself seems to impact DNA repair. Several labs have shown the formation of DNA:RNA hybrids around DSBs in different eukaryotes, either dependent on previous transcription^13,14^ or upon de novo transcription of the broken chromatin^15,16^. The relevance of such RNA molecules is still under debate, with both pro- and anti-repair effects ascribed to them^9^.

An important co-transcriptional RNA modification that, so far, has not been extensively studied in its putative relationship with DNA repair and the response to DNA damage is RNA editing. This process alters RNA sequences by the action of specific deaminases that convert one base into another. Every mammalian transcript can be subjected to RNA editing^17–19^. RNA editing can be classified into several categories^20^, including adenosine-to-inosine (A-to-I) deamination, which is accomplished by a family of RNA-specific adenosine deaminases known as ADARs^18,19^. This family is formed by ADAR1, ADAR2 (also known as ADARB1), and ADAR3; however, only ADAR1 and ADAR2 have been shown to present catalytic activity. A-to-I deamination is the most abundant form of RNA-editing in mammals and defects in this process are associated with human diseases, such as disorders of the central nervous system^21^ or pediatric astrocytomas^22^. Only limited information has been published regarding the connection of A-to-I editing and DNA damage, albeit at least the mRNA of NEIL1, has been shown to be re-coded by ADAR1 to alter its enzymatic properties^23^. Moreover, A-to-I editing has been proposed to be involved in the pathogenesis of cancer^24,25^.

Here, we show that the general pattern of ADAR2-mediated A-to-I editing changes upon DSB formation. Such changes depend on the DDR, specifically the ATR kinase and the resection protein CtIP. As a consequence, ADAR2 is required for the maintenance of genomic integrity and, specifically for DNA end resection and HR. Strikingly, mRNAs from either resection-related or recombination-related genes are not affected by ADAR2. Instead, ADAR2 role in resection is related to its ability to edit DNA:RNA hybrids. Not only do such structures increase when ADAR2 is depleted, but this protein physically and functionally interacts with the BRCA1-SETX complex for this role.

## Results

### RNA editing changes after DNA damage

**Fig. 1:**
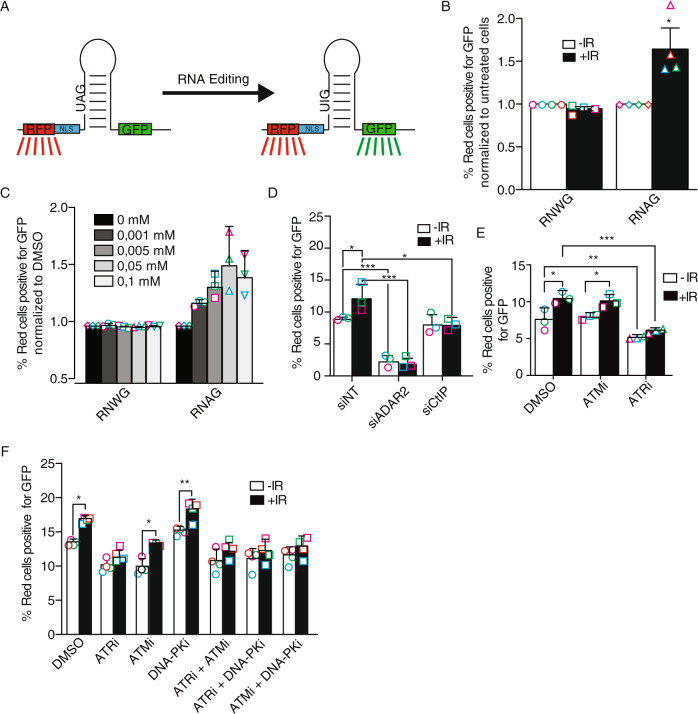
DNA damage increases RNA editing. **A** Scheme of the RNAG editing system. A bi-cistronic mRNA containing the RFP and GFP sequences is produced. The presence of a stop codon impedes the expression of the GFP ORF, except when the adenine is edited to inosine. The presence of a secondary structure containing such stop codon allows its recognition and deamination of the adenine by ADAR proteins. **B** DNA damage-induced RNA editing. The plot shows the percentage of cells bearing the RNAG reporter or the constitutively edited RNWG system that express both the RFP (red cells) and GFP (green cells). Cells were either irradiated (+IR; 10 Gy; black bars) or mock-treated (–IR; white bars) and incubated for 12 h. The percentage of green cells over 10.000 red cells were analyzed on BD FACSAriaTM using FACSDiva v5.0.3 software. For each reporter, the ratio of green and red cells was normalized with the untreated conditions. Statistical significance was determined with a two-tailed paired Student’s *t*-test. **C** Same as **B**, but cells were treated with the indicated concentration of camptothecin (CPT). **D** Cells bearing the RNAG reporter were transfected with the indicated siRNAs and irradiated or not, and the percentage of red cells that were also green is plotted. Statistical significance was determined with a two-way ANOVA. **E** Same as **D**, but cells were pretreated for 2 h with 10 μM of inhibitors of ATM (ATMi), ATR (ATRi), or DMSO as control, previous to the irradiation. Cells were collected to check for editing levels 10 h after irradiation. The inhibitors were kept for the duration of the experiment. Statistical significance was determined with a two-way ANOVA. **F** Same as **E**, but, cells were also treated with DNA–PK inhibitor (DNA–PKi), as well as the double combinations of the ATM, ATR, and DNA–PK inhibitors, as indicated. Statistical significance was determined with a two-way ANOVA. The average and the standard deviation of the medians of four (panels B and F) or three (panels C–E) independent experiments are shown. Each individual replica is marked with a colored symbol. One, two, or three asterisks represent *p* < 0.05, *p* < 0.01, or *p* < 0.001, respectively. Actual *p*-values can be found in the Source data file. Only biological relevant comparisons are shown.

As previously mentioned, crosstalk between RNA metabolism and DNA repair has been extensively documented^9^, but a connection between DNA repair and RNA editing has not been extensively analyzed. Thus, we wanted to study whether the appearance of DNA damage had any effect on RNA editing. In order to explore this possibility, we used a previously published reporter system (RNAG) that measures levels of RNA editing using the accumulation of the fluorescent proteins GFP and RFP^26^. This system bears both the RFP and GFP ORFs in a single transcript, with a stop codon between them (Fig. 1A). So, cells bearing such reporter express RFP constitutively, but GFP is only produced if an RNA editing event changes the A of the stop codon to an I (Fig. 1A)^26^. Therefore, the number of red cells that are also green indicates the efficiency of RNA editing. As a control to discard other effects non-related to the editing on this system, we used the RNWG control reporter, in which the stop codon is pre-edited, so all cells bearing the construct fluoresce, indeed, in red and green^26^. In U2OS cells stably transfected with the reporter, we observed that DNA damage induced by ionizing radiation increased GFP expression by 50% specifically in the RNAG reporter and not in the RNWG control, in agreement with a DNA damage stimulation of RNA A-to-I editing in this system (Fig. 1B). Similar results were obtained when using the DNA damage-inducing drug camptothecin (Fig. 1C), where we could observe a dose-dependent effect on RNA editing stimulation. One possibility is that DNA damage induces the accumulation of the A-to-I editing machinery, namely ADAR1 and ADAR2 enzymes, thus increasing this process. However, neither of these proteins was upregulated, but were slightly downregulated, upon exposure to IR (Supplementary Fig. 1A). Then, in order to confirm this was a canonical induction of RNA editing, we depleted the A-to-I editing machinery. To choose which member of the ADAR family to downregulate, we revisited the data we obtained in a previous genome-wide screening for factors that unbalance the choice between DSB repair pathways^27^. Interestingly, both ADAR1 and ADAR2, but not the catalytically inactive ADAR3, skewed DSB repair towards end-joining (Supplementary Figure 1B), and this was not due to changes in the cell cycle (Supplementary Fig. 1C). However, the effect was more prominent and clearer upon ADAR2 depletion. Indeed, downregulation of ADAR2 severely compromised both the basal and the DNA damage-induced expression of the GFP in the RNAG (Fig. 1D; for ADAR2 depletion efficiency see Supplementary Fig. 2A), but, as expected, not in the RNWG control reporter (Supplementary Fig. 2B).

To better understand this phenomenon, we decided to look for DDR factors that affected the DNA damage-induced RNA editing. Recently, we have found that CtIP, a core DNA end resection factor that is also required for ATR activation, plays additional roles in DNA damage-induced RNA splicing^28^. Interestingly, we could see that CtIP downregulation specifically eliminated the DNA damage-dependent induction of RNA editing without affecting the basal levels (Fig. 1D). Again, CtIP depletion did not alter GFP levels in the control RNWG system (Supplementary Fig. 2B). We could also complement this effect with the expression of siRNA-resistant flag-tagged CtIP in CtIP depleted cells, to the same extent as the control cells transfected with a non-targeting siRNA, even though overexpression of FLAG-CtIP on its own reduced the intensity of this DNA damage-induced phenotype (Supplementary Fig. 2C).

The general response to DNA damage is mainly controlled by the activation of two related apical kinases, ATM and ATR^1^. Thus, we also tested if any of them was required for the induction of RNA editing upon irradiation. Interestingly, ATM inhibition did not affect DNA damage-induced RNA editing, while ATR inhibition decreased the DNA damage-induced editing increase (Fig. 1E). This agrees with the notion that ATR and CtIP act on the same branch of the DNA damage checkpoint in response to DSBs^29^. The lack of response with the ATM inhibitor could be explained by a compensation by another member of the PIKK family, most likely DNA-PK. Along those lines, the ATR effect could also be affected by this phenomenon. Thus, we repeated the experiment with ATM, ATR, and DNA-PK inhibitors in different combinations (Fig. 1F). As shown, inhibition of ATR suppressed the DNA damage-induction of RNA editing, regardless of the presence of the inhibitors of ATM or DNA-PK. Interestingly, chemical inhibition of DNA-PK showed a limited increase in the basal levels of RNA editing, but importantly the exposure to DNA damage still provoked a hyperactivation of the process. Notably, concomitant inhibition of both DNA-PK and ATM abolished the induction of RNA editing caused by irradiation. Thus, it seems that those two kinases could have an overlapping role in this phenomenon.

### ADAR2 depletion causes genomic instability and DNA damage sensitivity

**Fig. 2:**
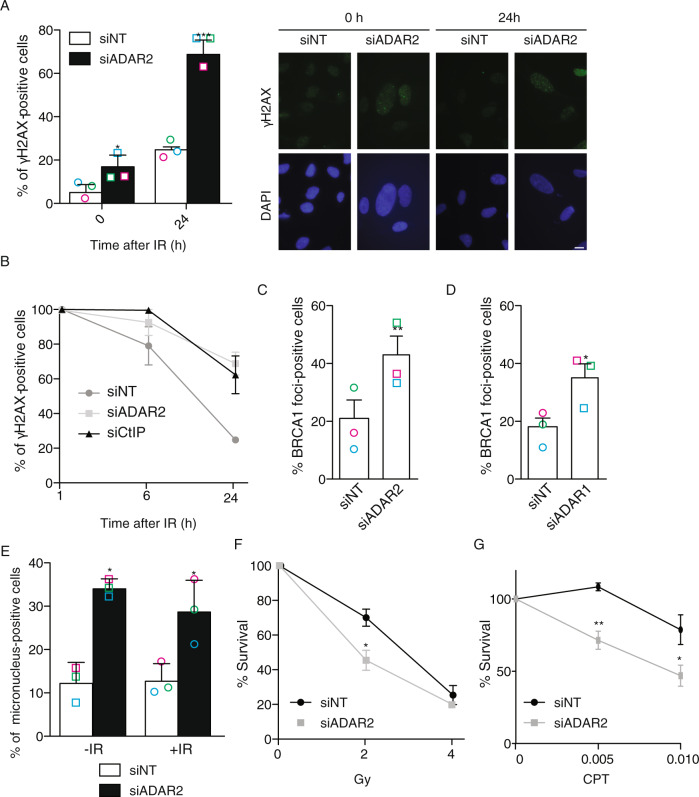
ADAR2 depletion causes genetic instability and DNA repair defects. **A** Percentage of cells positive for γH2AX foci upon spontaneous accumulation (0 h) or 24 h post irradiation with 10 Gy in U2OS cells transfected either a siRNA against ADAR2 or with control siNT. Quantification is shown on the left. A representative image is shown on the right. ﻿Scale bars represent 10 µm. **B** Repair kinetics is shown as the disappearance of γH2AX foci 1, 6, and 24 h post irradiation with 10 Gy in U2OS cells transfected either a siRNA against ADAR2, CtIP or with control siNT. **C** Percentage of spontaneous BRCA1 foci-positive cells in cells transfected with either a siRNA against ADAR2 or with control siNT. The average and SD of three independent experiments is shown. **D** Same as **C** but in cells depleted for ADAR1. **E** Percentage of cells positive for micronucleus without exposure to DNA damage (-IR) or 24 h post irradiation with 10 Gy (+IR) in U2OS cells transfected either with a siRNA against ADAR2 or with control siNT. Other details as in (**A**). **F** Clonogenic assays of U2OS cells depleted with a siRNA against ADAR2 or with control siNT after treatment with different doses of IR. Other details as in (**A**). **G** Same as **F** but cells treated with camptothecin (CPT; μM; right). In all panels, the average and SD of three independent experiments are shown and statistical significance was determined with a two-tailed paired Student’s *t*-test. Each individual replica is marked with a colored symbol. One, two, or three asterisks represent *p* < 0.05, *p* < 0.01, or *p* < 0.001, respectively. Actual *p*-values can be found in the Source data file. Only biological relevant comparisons are shown.

To understand the consequences of reduced A-to-I RNA editing for genomic stability, we decided to globally reduce such RNA modifications. Based on our previous data with ADAR2 (Fig. 1D and Supplementary Fig. 1B), we decided to use the downregulation of this protein as a tool to reduce A-to-I RNA editing. Strikingly, and in agreement with a role in maintaining genomic stability, the depletion of ADAR2 impaired DSB repair, measured as the presence of γH2AX foci 24 h after irradiation. Spontaneous DNA damage accumulated in the absence of any exogenous genotoxic agent in ADAR2-depleted cells (Fig. 2A). Confirming a DSB repair impairment, the disappearance of γH2AX foci upon exposure to ionizing radiation was delayed (Fig. 2B). Indeed, repair levels of DSBs at 6 and 24 h after irradiation in ADAR2-depleted cells were similar to those observed after downregulation of the critical repair factor CtIP (Fig. 2B). Interestingly, and in agreement with an increased burden of spontaneous DNA damage, in the absence of ADAR2, we observed a significant increase of BRCA1 foci in cells unchallenged with any genotoxic agent (Fig. 2C). A similar effect was observed upon ADAR1 downregulation (Fig. 2D). Furthermore, micronuclei accumulated at high levels in ADAR2-depleted cells, regardless of the exposure to an external source of DNA damage (Fig. 2E). Finally, and confirming the role of ADAR2 in DNA repair and the maintenance of genomic stability, its depletion rendered cells hypersensitive to DSBs-inducing agents such as ionizing radiation or camptothecin (Fig. 2F, G).

### ADAR2 depletion affects DNA repair pathway choice

**Fig. 3:**
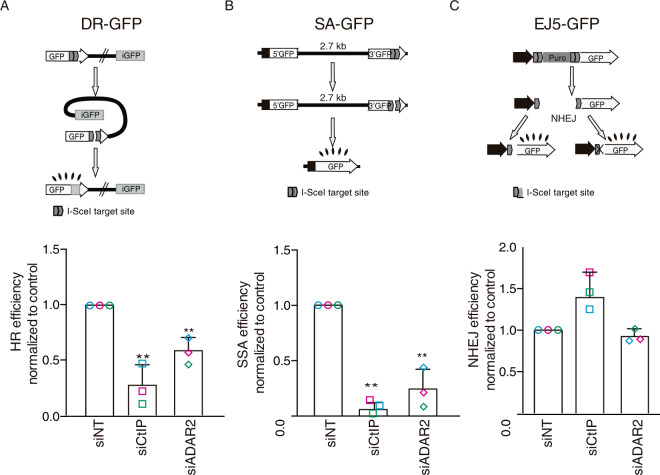
ADAR2 depletion affects homologous recombination. **A** Effect of ADAR2 depletion in the DR-GFP reporter. A scheme of the reporter is shown on the top. Induction of a DSB using I-SceI meganuclease renders GFP-positive cells when the donor repeat (iGFP) is used in a gene conversion event. The efficiency of classical recombination (HR) was calculated as the percentage of GFP-positive cells in response to I-SceI expression upon downregulation of the indicated genes and normalized with the control. The average and standard deviation of at least three independent experiments are shown. **B** Same as **A** but using the Single Strand Annealing (SSA) reporter SA-GFP (top). In this case, the induction of a DSB located between two repeats in direct orientation will render GFP-positive cells only when intramolecular SSA takes place. **C** Same as **A** but using the non-homologous end-joining (NHEJ) reporter EJ5-GFP (Top). In this case, two I-SceI-induced DSBs could be repaired by conservative or mutagenic NHEJ granting the accumulation of functional GFP. In all panels, the average and SD of three independent experiments are shown and statistical significance was determined with a two-tailed paired Student’s *t*-test. Each individual replica is marked with a colored symbol. One, two, or three asterisks represent *p* < 0.05, *p* < 0.01, or *p* < 0.001, respectively. Actual *p*-values can be found in the Source data file. Only biological relevant comparisons are shown.

Next, we decided to test a possible requirement of A-to-I RNA editing for DSB repair. As mentioned, ADAR2 depletion skewed the balance between HR and NHEJ towards the latter (see ref. ^27^and Supplementary Fig. 1B), suggesting that recombination might be compromised. Indeed, both RAD51-dependent gene conversion (GC) and RAD51-independent Single Strand Annealing (SSA), two types of homology-dependent repair (HDR), were reduced in cells downregulated for ADAR2 (Fig. 3A, B). In stark contrast, there was no impact on NHEJ efficiency (Fig. 3C), arguing that ADAR2 was particularly required for HR. The cell cycle is a major regulator of DSB repair pathway choice, as HR is limited in G1. However, the observed HR defect was not caused by an accumulation of G1 cells (Supplementary Fig. 1C). Thus, we conclude that ADAR2 facilitates repair by HR.

### DNA resection requires ADAR2 editing activity

**Fig. 4:**
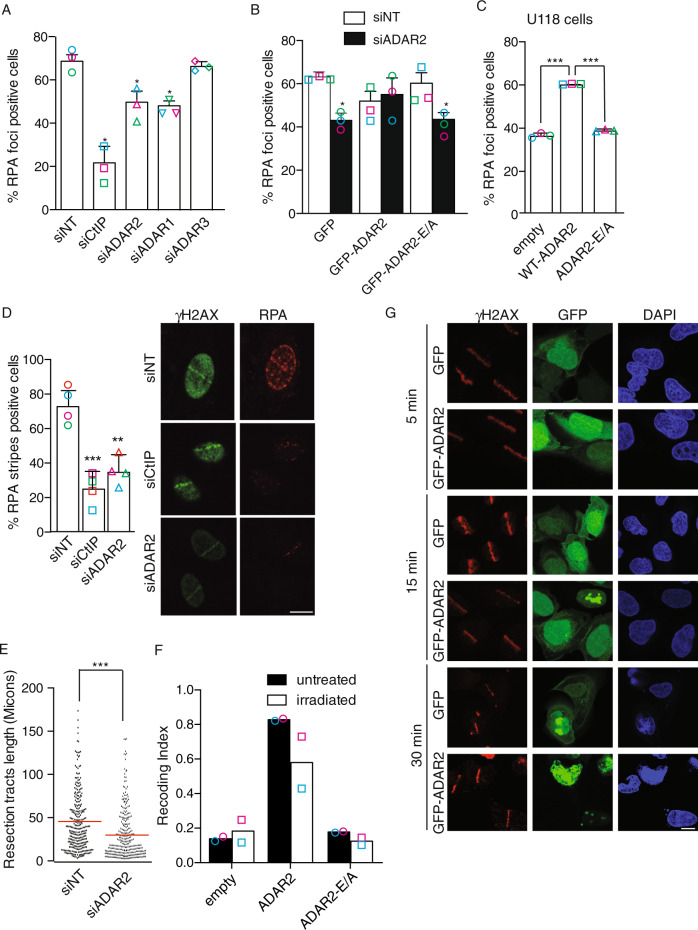
ADAR depletion impairs in DNA resection. **A** DNA resection proficiency after 10 Gy of irradiation in U2OS cells measured as the percentage of RPA foci-positive cells in cells transfected either with siRNAs against ADAR1, ADAR2, ADAR3, CtIP or with control siNT. The average and SD of three independent experiments are shown. Each individual replica is marked with a colored symbol. Significance was determined by two-tailed Student’s *t*-test comparing each condition to siNT cells. *P < 0.05. Actual *p-*values can be found in the Source data file. Representative images of the experiments are shown on Supplementary Fig. 3A. **B** DNA resection proficiency measured as the percentage of RPA foci-positive cells in U2OS cells expressing either GFP-ADAR2 wild type or a catalytically dead version of the protein (ADAR2 E/A) transfected either with a siRNA against the 3'UTR of ADAR2 (black boxes) or a control siNT-UTR (white boxes). Each individual replica is marked with a colored symbol. Significance was determined by two-tailed Student’s *t*-test comparing each condition to siNT cells. *P < 0.05. Actual *p*-values can be found in the Source data file. Other details as in (**A**). **C** RPA foci formation upon in U118 cells treated with 10 Gy of radiation in cells expressing either GFP, GFP-ADAR2 wild type or a catalytically dead version of the protein. Each individual replica is marked with a colored symbol. Significance was determined by two-tailed Student’s *t*-test. ***P < 0.001. Actual *p*-values can be found in the Source data file. Other details as in (**A**). **D** DNA resection proficiency was measured as RPA stripes-positive cells upon laser microirradiation in cells transfected either an siRNA against ADAR2, CtIP or with control siNT. The average and SD of four independent experiments are shown. Each individual replica is marked with a colored symbol. Representative images of the experiments are shown on the right. Scale bars represent 10 µm. Significance was determined by two-tailed Student’s *t*-test comparing each condition to siNT cells. **P < 0.01; ***P < 0.001. Actual *p*-values can be found in the Source data file. Other details as in (**A**). **E** Resection length measured with the SMART assay using DNA fibers extracted from U2OS downregulated for ADAR2. A non-target siRNA (siNT) was used as control. One out of three representative experiment with similar results is shown. Significance was determined by two-tailed Student’s *t*-test comparing. ***P < 0.001. Actual *p*-values can be found in the Source data file. Other details as in (**A**). **F** RNA sequencing of U118 cells complemented with a plasmid bearing wild type ADAR2, catalytically dead ADAR2 E/A or the empty vectors in untreated conditions (black bars) or upon exposure to 10 Gy of ionizing radiation (white bars) was used to analyze the changes in RNA sequence of codons known to be edited by ADAR2. The Recoding Editing Index (REI) was reported as percentage. The average of two independent experiments is shown. Each individual replica is marked with a colored symbol. **G** U2OS cells bearing GFP-ADAR2 or GFP, as a control, were micro-irradiated using a laser as described in the methods section. Cells were fixed at the indicated time points and the presence of γH2AX (red) or ADAR2 (green) at lasers stripes was analysed. Representative images out of three independent experiments are shown. Scale bars represent 10 µm.

Due to its effect in recombination, we hypothesized that ADAR2 might have a role in the common, early steps of the homology-dependent repair pathways, namely in DNA end resection. To test this idea, we first studied RPA foci formation upon ionizing radiation in ADAR2-depleted cells. RPA is an ssDNA binding complex that accumulates at DNA breaks as a direct consequence of DNA end resection^4,5,30^. Thus, the percentage of RPA foci-positive cells is the standard readout of resection in mammalian cells. Depletion of ADAR2 in U2OS cells caused a significant defect in resection, though less pronounced than that observed with the downregulation of the key resection factor CtIP (Fig. 4A and Supplementary Fig. 3A). For representative images of the experiment see Supplementary Fig. 3A. The same resection impairment was also observed upon depletion of ADAR2 in the HeLa cells (Supplementary Fig. 3B). Strikingly, similar results were observed upon depletion of ADAR1, but not the catalytically dead member of the ADAR family, ADAR3, thus suggesting that active RNA editing is required for DNA end resection (Fig. 4A and Supplementary Fig. 3A).

To confirm that the observed phenotype in DNA resection was truly due to the reduction of ADAR2 levels and not to an indirect off-target effect, we studied RPA foci formation in cells bearing siRNA-resistant, GFP-tagged variants of ADAR2. Indeed, the resection impairment caused by depletion of ADAR2 was rescued by wild-type GFP-ADAR2 (Fig. 4B and Supplementary Fig. 3C). Importantly, this rescue was not observed with the expression of a catalytically dead version of the protein (GFP-ADAR2E-A) (Fig. 4B), arguing that ADAR2 deaminase activity was required for processive resection. Moreover, we could reproduce the resection defect in U118 cells^31^, a glioblastoma cell line that is defective for ADAR2 expression, when compared with the same cells complemented with a wild-type copy of the gene, but not a catalytically dead mutant (Fig. 4C).

To validate these observations, we analyzed recruitment of RPA to DSBs by other means. U2OS cells depleted for ADAR2, or CtIP as a control, were laser micro-irradiated and immunostained with antibodies against RPA and γH2AX to identify the irradiated areas. The percentage of γH2AX-positive stripes that were co-stained by RPA was determined (Fig. 4D). In agreement with our previous results, depletion of ADAR2, or CtIP, significantly diminished the presence of RPA at the irradiated areas. Finally, in order to analyze in more detail the resection defect related to ADAR2 depletion and to investigate whether only resection initiation was impaired or if resection processivity was also compromised, we used SMART, a high-resolution technique that measures resection in individual DNA fibres^32,33^. As seen in Fig. 4E, the length of ssDNA fibres formed during the resection process was reduced upon ADAR2 depletion. Again, similar results were observed upon ADAR1 downregulation (Supplementary Fig. 3D), reinforcing the connection between A-to-I editing and DNA end resection.

Altogether, these results confirm that RNA editing by ADAR proteins facilitates DNA end resection at DSBs.

The role of ADAR2 on resection does not rely on the recoding of mRNAs that encode resection factors

Next, we wondered how this RNA editing activity might be needed for DNA end processing. We studied the recruitment DSB repair factors, such as 53BP1, BRCA1, and CtIP, to DNA damage foci shortly after DNA damage induction upon ADAR2 depletion. Notably, neither of them was affected in cells exposed to ionizing radiation or laser micro-irradiation (Supplementary Fig. 4A–C). Then, we wondered if ADAR2 was specifically editing mRNAs that code for resection factors. To analyze this possibility, we exposed the ADAR2-defective cell line U118 to ionizing radiation or mock treatment, isolated total RNA, and sequenced it. U118 cells complemented with either wild-type ADAR2 or a catalytically dead mutant were also processed in parallel. The levels of ADAR2 mRNA in the different samples are shown in Supplementary Fig. 4D. As expected, U118 cells complemented with wild-type ADAR2 showed a higher efficiency in editing the coding codons of known ADAR2 targets, expressed as the weighted average over all known recoding sites, known as the REI^34^. Instead, non-complemented U118 cells showed little recoding editing, regardless of whether or not exposed to ionizing radiation (Fig. 4F). Equally expected, the expression of a catalytically dead enzyme also showed almost no recoding of mRNAs (Fig. 4F), despite the fact that such variant was expressed almost 3 times more than the wild-type ADAR2 (Supplementary Fig. 4D). Thus, only the expression of catalytically active enzyme led to the expected ADAR2-dependent recoding due to editing of specific codons (Fig. 4F). Interestingly, although some specific coding codons were edited more efficiently upon irradiation than in mock-treated cells, we observed a general decrease in the recoding editing efficiency of known ADAR2 substrates upon exposure to DNA damage (Fig. 4F and Supplementary Data 1). Moreover, we could not observe any change in the editing of mRNA from genes that code for recombination or resection factors either in cells exposed to DNA damage or in undamaged cells (Supplementary Table S1). Thus, we conclude that the role of ADAR2 in resection and recombination does not rely on changes in the sequence or expression of specific mRNAs of bona fide DNA repair factors. To integrate our data, in which we observed a general ADAR2-mediated increase in RNA editing in the reporter (Fig. 1) accompanied by a general reduction of its activity on known targets (Fig. 4F), we hypothesized that upon DNA damage ADAR2 is mobilized to induce RNA editing at new sites. Such re-distribution could mean that at least a fraction of the protein would localize at sites of DNA-DSBs. To test this idea, we analyzed the presence of GFP-tagged ADAR2 at damaged chromatin at different time points by laser micro-irradiation (Fig. 4G). In agreement with our idea, a fraction of GFP-ADAR2 was readily recruited to sites of DNA damage as early as 5 minutes after laser micro-irradiation, something not observed in cells expressing only GFP as a control. In fact, 50% of cells showed colocalization between γH2AX and ADAR2 stripes. Interestingly, such recruitment seemed transient, as it was no longer observed 30 min after irradiation. This recruitment pattern agrees with the idea that ADAR2, or at least a fraction of the protein, changes its substrates and is channeled towards RNAs at the sites of broken chromatin to play a role in the early steps of DSB repair.

### ADAR2 facilitates resection over DNA:RNA hybrids

**Fig. 5:**
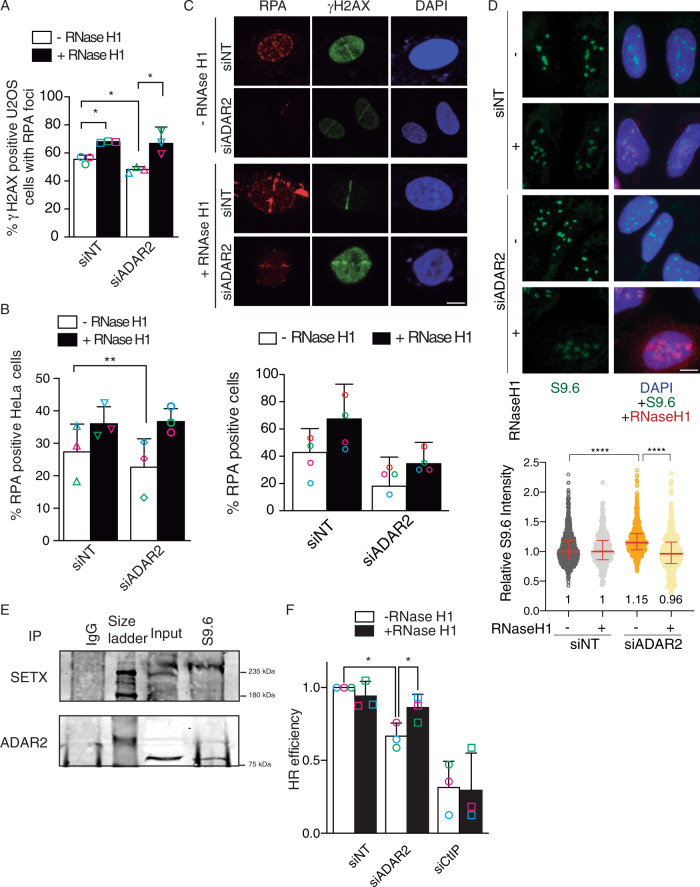
The connection of DNA resection defect with R-loop increase. **A** DNA resection proficiency measured as the percentage of RPA foci-positive cells after 1 h of 10 Gy of irradiation in cells U2OS transfected either with a siRNA against ADAR2 or with control siNT and transfected either with RNaseH1 overexpression plasmid (black) or with the control empty plasmid (white). The plot shows the percentage of cells positive for RPA foci and the average and standard deviation of at least four independent experiments. For each replicate, at least 200 cells were measured. The average and standard deviation of three independent experiments is shown. Each individual replica is marked with a colored symbol. Significance was determined by two-tailed Student’s *t*-test comparing each condition to siNT cells. *P < 0.05. Actual *p-*values can be found in the Source data file. Other details as Fig. 4A. **B** Same as A, but in HeLa cells. The average and standard deviation of three independent experiments is shown. Each individual replica is marked with a colored symbol. Significance was determined by two-tailed Student’s *t*-test comparing each condition to siNT cells. **P < 0.01. Actual *p*-values can be found in the Source data file. **C** HeLa cells were transfected with the indicated siRNAs and plasmids and micro-irradiated with a laser to induce DNA damage. Representative images are shown on top. Scale bars represent 10 µm. The percentage of cells positive for RPA recruitment to DSB are plotted below the images. The graph shows the average and standard deviation of four independent experiments. At least 20 cells per replica were studied and the number of stripes was analyzed using FIJI software. **D** Accumulation of RNA–DNA hybrids in ADAR2-depleted cells. U2OS cells transfected with siNT and siADAR2 and bearing the pcDNA3-RNaseH1 or pCDNA3 empty vector were immunostained to detect DNA:RNA hybrids using the S9.6 antibody. Relative S9.6 signal intensity per nucleus in U2OS cells with or without overexpression of RNaseH1 was calculated (bottom). The median with interquartile range obtained from three independent experiments for each population is shown. Statistical significance was calculated using a two-sided Mann–Whitney *U* test. Actual *p*-values can be found in the Source data file. One significant experiment out of three is shown. Scale bars represent 10 µm. **E** Protein samples from U2OS cells were immunoprecipitated using the anti-DNA:RNA hybrid S9.6 antibody or a non-related IgG as a control. Inputs and immunoprecipitates were resolved in SDS-PAGE and blotted for ADAR2 and Senataxin, as indicated. A representative western blot, out of three independent replicas, is shown. Source data are provided in the Source data file. **F** Effect of RNaseH1 overexpression in the ADAR2-mediated impairment of homologous recombination (HR). U2OS cells bearing the DR-GFP reporter were transfected with either a siRNA against ADAR2 or with control siNT and either with RNAseH1 overexpression plasmid (white) or with the control empty plasmid (black). The efficiency of classical recombination was calculated as the percentage of GFP-positive cells in response to I-SceI expression upon down-regulation of the indicated genes and normalized with the control. The average and standard deviation of three independent experiments are shown. Each individual replica is marked with a colored symbol. Significance was determined by paired two-tailed Student’s *t*-test comparing each condition to siNT cells. *P < 0.05. Actual *p*-values can be found in the Source data file. Other details as in Fig. 3A.

We hypothesized that the ADAR2-depletion related phenotype in DNA resection could be caused by a direct effect of ADAR2 on an RNA molecule located in the vicinity of the break that would act as a physical barrier for DNA end processing. The presence of DNA:RNA hybrids close to DSBs has been documented from yeast to mammals, both as pre-existing R-loops, R-loops formed as a consequence of breaks in transcribed regions or as DNA:RNA hybrids resulting from de novo transcription of resected DNA ends^9,13,16,35–37^. The actual effect of such DNA:RNA hybrids in resection is controversial, with both pro- and anti-resection effects described^9,16,38^. Importantly, ADAR2 has been proposed to recognize and edit DNA:RNA hybrids in vitro^39^. In order to define whether ADAR2 involvement in DNA end resection depended on the presence of DNA:RNA hybrids, we repeated the resection assay in the presence or absence of ectopically overexpressed RNaseH1, an enzyme that degrades the RNA moiety of such structures^40^. ADAR2 depletion and RNaseH1 overexpression efficiency are documented in Supplementary Fig. 5A. Strikingly, the overexpression of RNaseH1 in U2OS reverted ADAR2 resection phenotype as measured by RPA foci accumulation (Fig. 5A and Supplementary Fig. 5B). Indeed, the mere overexpression of RNaseH1 facilitated RPA foci formation even in cells transfected with a control siRNA, arguing that DNA:RNA hybrids act generally as physical barriers for the resection process, and that ADAR2 helps overcome such roadblocks. To confirm this finding, we studied the recruitment of RPA to damaged chromatin in HeLa cells depleted of ADAR2 and overexpressing RNaseH1. Again, such overexpression rescued the resection phenotype of ADAR2 depletion (Fig. 5B). The same was observed, albeit only partially, when laser micro-irradiation experiments were performed (Fig. 5C). In none of those cases, the increase in RPA-positive cells was due to changes in cell cycle profile when the RNaseH1 was overexpressed (Supplementary Fig. 5C). Then, to assess whether ADAR2 helped remove DNA:RNA hybrids, we analyzed the accumulation of such structures by immunofluorescence using the DNA:RNA hybrid-specific antibody S9.6^41^. As shown in Fig. 5D, depletion of ADAR2 increases the nuclear signal with that antibody. To rule out the contribution of other nucleic acid structures to the increase in the S9.6 signal, we overexpressed RNaseH1 and observed a significant reduction in the staining (Fig. 5D). Of relevance, ADAR2 has been previously found to be a part of the so-called “DNA:RNA hybrid interactome”^42^. Therefore, we decided to test if ADAR2 could interact directly with DNA:RNA hybrids. Indeed, ADAR2 was specifically immunoprecipitated using the S9.6 antibody, in a similar fashion as Senataxin (SETX), a helicase described to dissolve such hybrids and that has been shown to be recruited to DSBs at transcribed regions^13^ (Fig. 5E). This immunoprecipitation was specific, as did not occur when a control antibody was used (Fig. 5E).

Taken together, our results suggest the ADAR2 effect in resection was caused by DNA:RNA hybrid accumulation. A prediction of this model is that the decrease in HR caused by ADAR2 downregulation should be suppressed by RNaseH1 overexpression. In fact, recombination was almost completely restored in ADAR2-depleted cells when such enzyme was ectopically expressed (Fig. 5F). Such effect was not due to a general increase of recombination mediated by RNaseH1 overexpression, as it was not observed either in CtIP depleted or control cells.

### Increase of DNA:RNA hybrids generally impairs DNA end resection

**Fig. 6:**
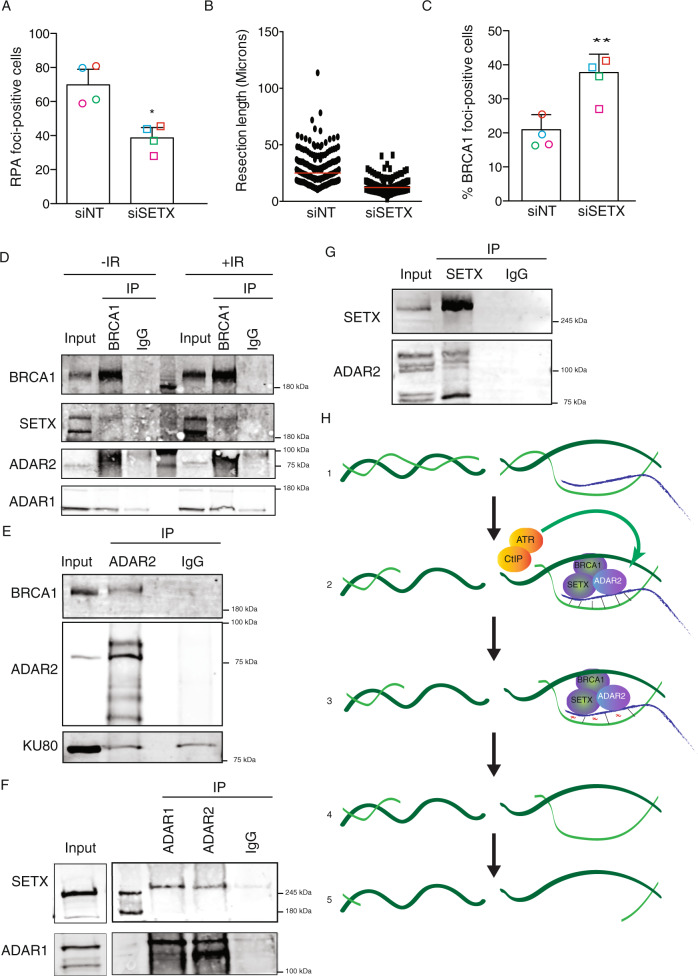
DNA:RNA hybrid stabilization impairs resection. **A** Percentage of RPA foci-positive cells in cells transfected either with a siRNA against SETX or with control siNT. The average and standard deviation of four independent experiments is shown. Each individual replica is marked with a colored symbol. Significance was determined by paired two-tailed Student’s *t*-test comparing each condition to siNT cells.*P < 0.05. Actual *p*-values can be found in the Source data file. Other details are as in Fig. 4A. **B** DNA resection proficiency is measured as the length of resected DNA with SMART in cells transfected either with a siRNA against SETX or with control siNT. Other details as in Fig. 4E. **C** Percentage of BRCA1 foci-positive cells in cells transfected either with a siRNA against SETX or with control siNT. The average and standard deviation of four independent experiments are shown. Each individual replica is marked with a colored symbol. Significance was determined by paired two-tailed Student’s *t*-test comparing each condition to siNT cells. **P < 0.01. Actual *p*-values can be found in the Source data file. Other details as in Fig. 1C. **D** Protein samples from U2OS cells were immunoprecipitated using an anti-BRCA1 antibody or a non-related IgG as a control, in cells irradiated (+IR; right) or not (-IR; left). Inputs and immunoprecipitates (IP) were resolved in SDS-PAGE and blotted for BRCA1, ADAR1, ADAR2, and Senataxin, as indicated. A representative western blot, out of four independent replicas, is shown. Source data are provided in the Source data file. **E** Protein samples from U2OS cells were immunoprecipitated using an anti-ADAR2 antibody or a non-related IgG as a control, in non-irradiated cells. Inputs and immunoprecipitates were resolved in SDS-PAGE and blotted for BRCA1, ADAR2, SETX and Ku80 as indicated. A representative western blot, out of three independent experiments, is shown. Source data are provided in the Source data file. **F** Protein samples from U2OS cells were immunoprecipitated using an anti-Senataxin antibody or a non-related IgG as a control, in non-irradiated cells. Inputs and immunoprecipitates were resolved in SDS-PAGE and blotted for Senataxin and ADAR2, as indicated. A representative western blot, out of three, is shown. Source data are provided in the Source data file. **G** Protein samples from U2OS cells were immunoprecipitated using an anti-SETX antibody or a non-related IgG as a control, in non-irradiated cells. Inputs and immunoprecipitates were resolved in SDS-PAGE and blotted for ADAR2 and SETX as indicated. A representative western blot, out of three independent experiments, is shown. **H** A schematic representation of how ADAR2 might help resection. **1** DNA:RNA hybrids might appear close to DSBs, either because they were already formed there or specifically formed upon DNA damage. Those hybrids will block resection progression. **2** The formation of some ssDNA by CtIP will activate the ATR branch of the checkpoint, that in turn will stimulate the activity of ADAR2 at DNA:RNA hybrids, including those close to DNA breaks. **3** ADAR2 activity will create mismatches in the DNA:RNA pairing (red tilde), facilitating the dissolution of those structures by SETX–BRCA1. **4**–**5** Once the DNA:RNA hybrids are eliminated, resection can proceed unimpeded.

Due to our observation that DNA:RNA hybrids hampered resection in the absence of ADAR proteins, we wondered if other enzymes involved in the removal of hybrids also affected DNA end resection, therefore this represented a more general phenomenon. We decided to test SETX, due to its aforementioned relationship with DSBs. So, we checked if its loss also affected RPA foci formation. Indeed, depletion of Senataxin also produced a DNA resection defect measured by RPA foci accumulation (Fig. 6A) and reduced the length of resected DNA (Fig. 6B). As seen for ADAR2 depletion, SETX downregulation also led to an increased burden of spontaneous DNA damage, measured as BRCA1 foci in unchallenged cells (Fig. 6C).

### ADAR proteins physically interact with Senataxin and BRCA1

Our data suggested a possible role of ADAR2 in R-loop resolution, which would be key for facilitating DNA end resection and HR. Hence, we wondered if ADAR2 might interact with known players in the homeostasis of R-loops that are also connected with DSB repair. Many different enzymes have been associated with the removal of R-loops. Among them, we decided to focus on the BRCA1–SETX complex. These two proteins have been shown to cooperate in the elimination of R-loops in the 3' end of many transcribed genes^43^. Moreover, both proteins play roles in DNA end resection, as it has been previously established for BRCA1^33^ and in this study for Senataxin (Fig. 6A–C). Thus, we tested whether ADAR proteins might physically interact with BRCA1 and Senataxin. Using antibodies against BRCA1, we confirmed the previously shown interaction of this protein with Senataxin^43^ and also established an interaction with ADAR1 and ADAR2 (Fig. 6D). An additional, independent co-immunoprecipitation experiment between ADAR2 and BRCA1 can be found in Supplementary Fig. 6A. These interactions were not stimulated by the presence of DNA damage, at least measured 1 h after irradiation (Fig. 6D). Also, by co-immunoprecipitations with different antibodies we could observe the reciprocal interaction of ADAR proteins with both BRCA1 and SETX (Fig. 6E–F). The possibility that ADAR immunoprecipitation could be bringing down any proteins accumulating at sites of DNA DSBs was excluded, since we could not observe an interaction of ADAR2 with the DNA-end binding protein Ku80, suggesting that those interactions were, indeed, specific. It is worth pointing out that these co-immunoprecipitations were performed in the presence of benzonase, thus they are not bridged by DNA. Moreover, immunoprecipitation using an antibody against SETX also allows the co-precipitation of ADAR2 (Fig. 6G). Additional, independent co-immunoprecipitation experiments between ADAR1, ADAR2, and SETX can be found in Supplementary Fig. 6B. Therefore, we could confirm that these proteins interact in a DNA damage-independent fashion, most likely to facilitate the removal of DNA:RNA hybrids globally.

## Discussion

mRNA post-transcriptional modifications are now being revealed as potent regulators of cellular metabolism that allow flexibility and adaptability to a changing environment^44^. Such chemical modifications of the mRNA can recode coding sequences, create or destroy splicing sites, affect RNA stability and structure, or directly interact with specific readers in order to recruit specific machinery affecting translation and/or mRNA localization. Therefore, those modifications affect virtually every aspect of the biological response of the cells. In fact, it has been recently shown that other base modifications of RNA happen at DNA:RNA hybrids and contribute to genomic stability^45^. Thus, it was reasonable to expect that A-to-I RNA editing would contribute to the global response to DNA damage. Indeed, our results support this notion, uncovering a bona fide role of A-to-I deamination by ADAR1 and ADAR2 in the maintenance of genomic integrity upon exposure to genotoxic agents. ADAR2-mediated RNA editing seems, indeed, completely upturned upon treatment with DNA damaging agents. Many of the codons in mRNAs that are usually targeted by ADAR2 decrease their editing, whereas specific codons on natural mRNAs and in other RNA species such as the RNAG reporter increase their editing. Based on these findings, we propose an RNA-editing DDR (REDAR) that is essential for DNA repair, and specifically for HR, contributing to the maintenance of genomic integrity.

Specifically, we postulate that upon the triggering of the DDR, REDAR is activated by ATR and CtIP. Then, ADAR2, and maybe ADAR1, is mobilized from its usual targets to new ones, most likely including DNA:RNA hybrids, in order to help with their dissolution. In this regard, ADAR2 action might have two non-mutually-exclusive outcomes; on the one hand, it can rapidly and transiently relocate to promote RNA editing at the sites of DSBs, aiding in the removal of DNA:RNA hybrids and facilitating DNA end resection; and on the other hand, it can increase the editing of a small fraction of yet-undisclosed mRNAs, whose role in the DDR should be clarified in further studies. Interestingly, it has been previously shown that ADAR action can alter the biochemical properties of some DNA repair enzymes^23^. We envision that the transcriptional inhibition induced by the DDR^46,47^ will cause a reduction of the normal co-transcriptional RNA editing^48^ and will free ADAR to act on alternative substrates such as the aforementioned DNA:RNA hybrids. We favor the idea that the editing itself would happen on the RNA moiety of the structure. However, by performing an in silico study we observed that cancer cells that overexpress ADAR2 are statistically more likely to accumulate this kind of mutations (Supplementary Fig. 7). Thus, it is also possible that the editing occurs in the DNA strand, as suggested in vitro^39^, albeit how frequently and whether this happens in the context of DNA repair is still unclear. Editing of the DNA strand would greatly increase the mutagenesis associated with HR, something that might be deleterious for the cells, but has been observed due to the action of C-to-U deaminases in cancer^49^. Interestingly, the accumulation of N6-methyladenosine modification on the RNA at DNA:RNA hybrids has also been shown to ease the dissolution of such structures^45^. As A-to-I editing is negatively influenced by m^6^A modifications^50,51^, the crosstalk between those two RNA post-transcriptional modifications during DNA repair and in response to DNA damage will be worth exploring further.

The mechanism by which ADAR2 is relocated in response to DNA damage is still far from clear. On the one hand, a passive model is possible, in which ADAR2 naturally recognizes and binds to DNA:RNA hybrids. It has been shown that DNA damage induces the formation of such structures and, in this scenario, the accumulation of hybrids would sequester ADAR2, reducing its availability to edit its usual targets. Indeed, using GFP-tagged versions of ADAR2 we could observe a re-localization to laser micro-irradiated stripes. However, such accumulation is very transient, and in a completely different timeframe of the changes in recoding we have also shown. So, this recruitment cannot fully explain the changes in editing we observed globally, arguing for the coexistence of both a global, genome-wide effect, and a site-specific role. It is even possible that, in this context, during REDAR, ADAR2 would not act specifically at DNA:RNA hybrids that are close to DSBs, but rather relocated to hybrids spread through the genome as a broad response to DNA damage. In this scenario, the accumulation at laser lines we observed would rely simply on the already known accumulation of hybrids at break sites. Indeed, the SETX–BRCA1 complex has been proposed to be important and recruited mainly to transcription termination sites, regardless of the presence of DNA damage^43^, thus again arguing with for putative role that does not require specific recruitment to DSBs. Alternatively, there could be an active, recruitment of ADAR2 to hybrids that sit specifically in the proximity of DSBs. The physical interaction with BRCA1, a well-established, bona fide member of the DDR, which is recruited to DSBs^52^ and Senataxin, that is also localized to damaged chromatin^13^, might favor this model. This idea is also supported by the fact that A-to-I editing at new sites is upregulated upon REDAR activation in an ATR-dependent manner, arguing for a DNA damage-dependent recruitment of ADAR2 to those new target sites. Interestingly, the ATR checkpoint is mainly active in S and G2, as it requires ssDNA for its activation that is produced by DNA end resection. This would explain why CtIP downregulation also decreases the DNA damage-induced increase of RNA editing in the RNAG system, simply reflecting the role of CtIP in the ATR activation upon formation of DSBs.

CtIP is linked to RNA metabolism in multiple ways. It affects RNA editing, as shown here, but also interacts with multiple RNA binding proteins^53^ that in turn are required for proper resection. Moreover, we have recently shown that CtIP controls the splicing of specific factors, in many cases facilitating the accumulation of specific alternative splicing forms upon exposure to DNA damage^54^. Additionally, CtIP not only interacts with BRCA1, which also affects RNA splicing, but also CtIP deficiency has been shown to promote the accumulation of DNA:RNA hybrids at sites of highly expressed genes^14^. Paradoxically, CtIP depletion reduces DNA:RNA hybrid accumulation dependent on de novo transcription of dilncRNA (damage-induced long non-coding RNAs) starting at DSBs^36^. Hence, CtIP depletion seems to increase R-loops that are produced as a consequence of the previous transcription and appears to decrease de novo production of diRNAs (DSB-induced small RNA), thus reducing the DNA:RNA hybrids formed after DNA damage. The data suggest that DNA resection, and specifically CtIP and BRCA1, are in close relationship with the general metabolism of RNAs, a reciprocal connection that is worth to continue exploring.

To integrate all our observations, we present a model in which ADAR2 (and most likely ADAR1), BRCA1, and SETX facilitate the removal of hybrids genome-wide even in the absence of DNA damage, but in a way that is stimulated by the DDR during REDAR. When exposed to a source of DSBs, the levels of DNA:RNA hybrids and R-loops increase, as previously described by other authors^13–16^, and many of them specifically accumulate at break sites. Although some authors argue that these structures might favor the repair process^38^, others favor a view in which they act as roadblocks for repair (for a review see ref. ^37^). This contradiction might simply stem from a differential effect of DNA:RNA hybrids depending on the timing of repair, i.e. very early events might need them but later they have to be eliminated, or the position of the hybrids, as discussed elsewhere^9,55^. Our data agree with the notion that at least some of them represent roadblocks for the progression of the repair machinery that has to be removed prior to repair (Fig. 6G-1). Such blocking effect of R-loops is well established for other DNA transactions such as transcription and replication^37,56^. We propose that, upon irradiation, a CtIP- and ATR-mediated global induction of A-to-I editing at DNA:RNA hybrids facilitate their removal both genome-wide and locally, at sites of DNA breaks, hence permitting the DNA end resection machinery to overcome the physical barrier represented by pre-existing or recently formed hybrids close to the DNA breaks (Fig. 6G–2). Mechanistically, we suggest that ADAR-mediated editing of DNA:RNA hybrids, an activity that has been confirmed in vitro^39,57^, might alter the sequence of the RNA strand creating ribo–inosine (rI) and the deoxiribose–thymine (dT) mismatches (Fig. 6G–3). The appearance of those mismatches will loosen up the interaction between the RNA and the DNA strand, facilitating the unwinding of the structure by the helicase activity of SETX (Fig. 6G–3,4), allowing the resection machinery to go through (Fig. 6G–4,5). Strikingly, ADAR proteins were first discovered in Xenopus as having developmentally regulated dsRNA unwinding activity in oocytes^58^, and later shown to rely on the modification of A–to–I in the RNA substrate, which modified the base-pairing properties and facilitated the melting of that RNA:RNA double-stranded structures^59^. Alternatively, rather than physically loosening the interaction between the DNA and RNA, A-to-I modification of the RNA might help the recruitment of proteins involved in R-loop removal or even the recruitment of bona fide resection factors. We propose that this alteration of the RNA moiety of the hybrids will happen at all R-loops scattered across the genome, even in the absence of DNA damage. But particularly at DSBs, ADAR2 will contribute to the processivity of resection over DNA:RNA hybrids, regardless of if these structures preceded or formed as a consequence of the break in its vicinity. Our model proposes a role of ADAR in DNA end resection and recombination that acts independently but not exclusively, of the recoding or regulation of expression of yet-undisclosed mRNAs. Interestingly, recently it has been shown that ADAR1 editing activity is required to eliminate R-loops at telomeric repeats in order to preserve genomic integrity^57^. In this case, ADAR1 has been proposed to edit mismatched hybrids formed by TERRAs and non-canonical telomeric repeats in order to facilitate its removal by RNaseH2, a phenomenon that is specific in non-ALT cancer cell lines. Thus, albeit with slightly different mechanisms, this confirms that RNA editing affects genomic integrity by regulating DNA:RNA hybrid elimination.

Strikingly, ADAR deficiency, and generally unbalance the levels of editing of the RNA, have been associated with the development of many different tumors^24,25,34,60^. Specific editing events have already been associated with glioblastoma^22,61^. However, in the light of this new role for ADAR2 in DNA repair and the DDR, it might be important to extend the connection of this protein with cancer and establish if it is linked to a particular genomic instability signature.

## Methods

### Cell lines and growth conditions

All cell lines were grown in DMEM (Sigma-Aldrich) supplemented with 10% fetal bovine serum (Sigma-Aldrich), 2 mM L-glutamine (Sigma-Aldrich), 100 units ml^−1^ penicillin, and 100 μg ml^−1^ streptomycin (Sigma-Aldrich). U2OS and U118-derived cell lines stably expressing GFP or GFP–ADAR2 plasmids^31^ were grown in a standard medium supplemented with 0.5 mg ml^−1^ G418 (Gibco, Invitrogen). Cells expressing RNWG and RNAG were grown in standard medium supplemented with 0.5 mg ml^−1^ G418 (Gibco, Invitrogen).

### siRNAs, plasmids and transfections

siRNA duplexes were obtained from Sigma-Aldrich or Dharmacon (Supplementary Table 1) and were transfected using RNAiMax Lipofectamine Reagent Mix (Life Technologies), according to the manufacturer’s instructions. RNWG and RNAG was a gift from Dr. Jantsch’s lab^26^. The GFP-ADAR2 and GFP-ADAR2 mutant (GFP-ADAR2-E/A-) plasmids were previously described^31^. RNaseH1 overexpression was achieved with the pCDNA3–RNAseH1 vector^62^ and pCDNA3 (Invitrogen) was used as a control. Plasmid transfection of U2OS cells was carried out using FuGENE 6 Transfection Reagent (Promega) according to the manufacturer’s protocol, with the exception of pCDNA3–RNaseH1 and pCDNA3 plasmids that were transfected using Lipofectamine 3000 (Invitrogen) according to the manufacturer’s instructions.

### HR and NHEJ analysis

U2OS cells bearing a single copy integration of the reporters DR-GFP (Gene conversion)^63^, SA-GFP (SSA)^64^, or EJ5-GFP (NHEJ)^64^ were used to analyze the different DSB repair pathways. In all cases, 50,000 cells were plated in 6-well plates in duplicate. One day after seeding, cells were transfected with the indicated siRNA and the medium was replaced with a fresh one 24 h later. The next day, each duplicate culture was infected with lentiviral particles containing I-SceI–BFP expression construct at MOI 10 using 8 µg/ml polybrene in 1.5 ml of DMEM. Then, cells were left to grow for an additional 24 h before changing the medium for fresh DMEM, 48 h after siRNA transfection, cells were washed with PBS, trypsinized, neutralized with DMEM, centrifuged for 5 min at 700 g, fixed with 4% paraformaldehyde for 20 min, and collected by centrifugation. Then, cell pellets were washed once with PBS before resuspension in 150 µl of PBS. Samples were analyzed with a BD FACSAria with the BD FACSDiva Software v5.0.3. Four different parameters were considered: side scatter (SSC), forward scatter (FSC), blue fluorescence (407 nm violet laser BP, Filter 450/40), green fluorescence (488 nm blue laser BP Filter 530/30). Finally, the number of green cells from at least 10,000 events positives for blue fluorescence (infected with the I-SceI–BFP construct) was scored. The average of both duplicates was calculated for each sample of every experiment. To facilitate the comparison between experiments, this ratio was normalized with siRNA control. At least four completely independent experiments were carried out for each condition and the average and standard deviation are represented.

### RNA editing assay in vivo

The cells were seeded in 60 mm plates and transfected with siRNAs 24 h later. The cells were irradiated with 10 Gy or treated with the indicated doses of CPT and incubated during 12 h before harvesting. In the experiments performed with protein inhibitors, the medium was exchanged 2 h before irradiation with fresh DMEM containing 10 μM ATMi (KU55933), 5 μM ATRi (ETP46464), 10 μM DNA–PKi (NU7441), or DMSO as control. After that, the medium was replaced, DSBs were induced with the indicated DNA damage agent, and cells were incubated for 10 h. Then, cells were harvested with trypsin, spun down at 500 g for 5 min and washed with PBS. The cells were fixed with 4% paraformaldehyde for 15 min at 4 °C in the dark and later rinsed and resuspended in 150 μl of PBS. Red and green fluorescence was measured on BD FACSAriaTM using FACSDiva v5.0.3 software as indicated in the above section.

### Clonogenic cell survival assays

To study cell survival after DNA damage, clonogenic assays were carried out seeding cells in 6-well plates at two different concentrations in triplicates. DSBs were produced by IR or by acute treatment with topoisomerase inhibitor camptothecin (CPT; Sigma). For IR, 250 and 500 transfected cells were seeded per well and, for drug treatments, 500 and 1,000 cells per well. The following day, cells were exposed to DNA damaging agents: 2 Gy, 4 Gy or mock treated or incubated for 1 h with 0.01, 0.05, or 0.1 μM CPT or vehicle (DMSO) as control. After two washes with PBS, a fresh medium was added and cells were incubated at 37 °C for 7–14 days to allow colony formation. Afterward, cells were stained and visualized in the solution of 0.5% Crystal Violet (Merck) and 20% ethanol (Merck). Once the colonies were stained, this solution was removed and plates were washed with water. The surviving percentage at each dose was calculated by dividing the average number of visible colonies in treated versus control (untreated or vehicle-treated) dishes.

### SDS-PAGE and western blot analysis

Protein extracts were prepared in 2× Laemmli buffer (4% SDS, 20% glycerol, 125 mM Tris–HCl, pH 6.8) and passed ten times through a 0.5 mm needle–mounted syringe to reduce viscosity. Proteins were resolved by SDS–PAGE and transferred to low fluorescence PVDF membranes (Immobilon-FL, Millipore). Membranes were blocked with Odyssey Blocking Buffer (LI-COR) and blotted with the appropriate primary antibody and infrared dyed secondary antibodies (LI-COR) (Supplementary Tables 2, 3). Antibodies were prepared in blocking buffer supplemented with 0.1% Tween-20. Membranes were air-dried in the dark and scanned in an Odyssey Infrared Imaging System (LI-COR), and images were analyzed with Image Studio software (LI-COR).

### Immunoprecipitation

U2OS cells or U2OS cells containing GFP or GFP-CtIP were harvested in lysis buffer (50 mM Tris–HCl, pH 7.4, 100 mM NaCl, 1 mM EDTA, 0.2 % Triton X-100, 1X protease inhibitors (Roche), 1X phosphatase inhibitor cocktail 1 (Sigma)) and incubated for 30 min on ice with Benzonase (90 U/ml), with the exception of the S9.6 IP in which samples were sonicated for 15 min. Protein extract (1 mg) was incubated at 4 °C with 10 μl of anti-BRCA1, anti-SETX, anti-ADAR2, or S9.6 antibody (Supplementary Table 2) or with an equivalent amount of IgG (Mouse or Rabbit) as the negative control. Afterward, extracts were incubated with magnetic protein A Dynabeads (Novex) overnight. Beads were then washed three times with lysis buffer, and the precipitate was eluted in 50 μl of Laemmli buffer 2x.

### Immunofluorescence and microscopy

For RPA, γH2AX, and BRCA1 foci visualization, U2OS cells knocked down for different proteins were seeded on coverslips. The cells were treated with 10 Gy ionizing irradiation and incubated for 1 h. Then, coverslips were washed once with PBS followed by treatment with Pre extraction Buffer (25 mM Tris–HCl, pH 7.5, 50 mM NaCl, 1 mM EDTA, 3 mM MgCl_2_, 300 mM sucrose, and 0.2% Triton X-100) for 5 min on ice. Cells were fixed with 4% paraformaldehyde (w/v) in PBS for 20 min. For 53BP1 foci, cells growing on coverslips were treated for 10 min on ice with methanol and 30 s with acetone. In all cases, following two washes with PBS, cells were blocked for 1 h with 5% FBS in PBS, co-stained with the appropriate primary antibodies (Supplementary Table 2) in blocking solution overnight at 4 °C or for 2 h at room temperature, washed again with PBS and then co-immunostained with the appropriate secondary antibodies (Supplementary Table 3) in blocking buffer. After washing with PBS and dried with ethanol 70% and 100% washes, coverslips were mounted into glass slides using Vectashield mounting medium with DAPI (Vector Laboratories). Images were acquired and analyzed using a Leica fluorescence microscope. The analysis of 53BP1 number foci formation was performed automatically using MetaMorph software.

### SMART (Single-Molecule Analysis of Resection Tracks)

SMART was performed as described^32^. Briefly, cells were grown in the presence of 10 μM BrdU for <24 h. Cultures were then irradiated (10 Gy) and harvested after 1 h. Cells were embedded in low-melting agarose (Bio-Rad), followed by DNA extraction. DNA fibers were stretched on silanized coverslips, and immunofluorescence was carried out to detect BrdU (Supplementary Table 2, 3). Samples were observed under a Nikon NI-E microscope, and images were taken and processed with the NIS ELEMENTS Nikon Software. For each experiment, at least 200 DNA fibers were analyzed, and the length of the fibers was measured with Adobe Photoshop CS4.

### Cell cycle analysis

Cells were fixed with cold 70% ethanol overnight, incubated with 250 μg ml^−1^ RNase A (Sigma) and 10 μg ml^−1^ propidium iodide (Fluka) at 37 °C for 30 min and analyzed with a FACSCalibur (BD). Cell cycle distribution data were further analyzed using ModFit LT 3.0 software (Verity Software House Inc).

### UV laser micro-irradiation

Cells were micro-irradiated using a wide-field Angström’s microscope (Leica) equipped with a Micropoint pulsed dye laser of 365 nm (Photonic Instruments, Inc.). The cells were seeded in 25 mm coverslips and cultured overnight in the presence of 10 μM BrdU before laser micro-irradiation. About 40–50 cells were micro-irradiated with one laser stripe per cell. For RPA study, cells were pre-permeabilized with CSK Buffer (10 mM PIPES, 300 mM sucrose, 100 mM NaCl, 3 mM MgCl_2_, 1 mM EGTA) for 10 min. Then, cells were fixed for 10 min with 3.6% formaldehyde, permeabilized with 0.1% Triton X-100 for 15 min, and blocked for 30 min with 5% bovine serum albumin (BSA) in phosphate-buffered saline (PBS). Antibodies (Supplementary Tables S3, S4) were diluted in 1% BSA in PBST (PBS containing 0.01% Tween-20) and incubated for 1 h. Coverslips were mounted using Vectashield mounting medium with DAPI (Vector Labs) and the slides were visualized in an LSM780 confocal microscope (Carl Zeiss) with an optical thickness of 0.9 μm. Quantitative analyses of the number of cells with foci or stripes were carried out in random areas using FIJI software. The number of stripes was quantified in 20–30 cells per preparation.

### RNA isolation, RNA sequencing, and in silico analysis

RNA extracts were obtained from cells using the RNeasy Mini kit (QIAGEN, 74104) according to the manufacturer’s instructions. The RNA was purified with a standard phenol:chloroform extraction followed by ethanol precipitation.

RNA concentration was quantified by measuring 260 nm absorbance using a NanoDrop ND-1000 spectrophotometer, and the quality of the sample was checked by running a 1% agarose gel and by RNA 6000 Nano assay on a 2100 Bioanalyzer (Agilent Technologies).

RNA-Seq data (76 bp strand-oriented reads generated from Illumina platform) were first processed for adapters trimming and low-quality reads filtering. Then, cleaned reads were mapped against reference human genome (hg19), transcriptome and dpSNP by HISAT2 v.2.0.1^65^, and only uniquely and concordantly mapped reads have been used for subsequent analyses. RNA editing analysis was performed using a specific Python tool, REDItools^66^, with default parameters for the detection of the RNA editing sites collected in the REDIportal database^67^. Recoding Editing Index was calculated as previously shown^34^.

### DNA:RNA hybrid detection

S9.6 (hybridoma cell line HB-8730) and RNaseH1 (15606-1-AP; Proteintech) immunofluorescence (IF) was performed 48 h after siRNA transfection. Cells were fixed with ice-cold methanol, blocked and subsequently incubated with the primary and secondary antibodies. Nuclei were stained with DAPI. The S9.6 signal in nucleoli was subtracted from the integrated nuclear S9.6 signal to perform the analysis. Immunofluorescence images were acquired using a Leica DM6000 wide-field microscope equipped with a DFC390 camera at x63 magnification using the LAS AF software (Leica). Microscopy data analysis was performed using the Metamorph v7.5.1.0 software (Molecular Probes).

### ICGC data retrieval and analysis

Mutations sets were retrieved from the International Cancer Genome Consortium (ICGC) Data Portal MALY-DE and CLLE-ES datasets. ADAR2 expression levels from each donor were obtained using the UCSC Xena web tool. Open-access somatic mutations information from each mutation set was obtained by comparing each mutation set with the latest release of the Aggregated Somatic Mutation VCF file by the ICGC using custom python scripts. The percentage of A to G and T to C mutations was calculated as the quotient between the number of A to G mutations and T to C mutations to the total number of mutations from each donor set.

### Statistical analysis

Unless specifically specified, statistical significance was determined with a Student’s *t*-test using PRISM software (Graphpad Software Inc.). Statistically significant differences were labeled with one, two, or three asterisks if *p* < 0.05, *p* < 0.01, or *p* < 0.001, respectively.

### Reporting summary

Further information on research design is available in the Nature Research Reporting Summary linked to this article.

## Supplementary information

Supplementary Information

Description of Additional Supplementary Files

Supplementary Data 1

Reporting Summary

## Data Availability

The data supporting the findings of this study are available from the corresponding authors upon reasonable request. RNA-sequencing data have been deposited Sequence Read Archive (SRA) database and are accessible through the SRA accession number PRJNA747125. Source data for the figures and supplementary figures are provided as a Source data file. Source data are provided with this paper.

## Source data

Source Data
