# Supplementary material for: ADAR-mediated RNA editing of DNA:RNA hybrids is required for DNA double strand break repair
Source: Nat Commun. 2021 Sep 17;12:5512. doi: 10.1038/s41467-021-25790-2 (PMC8448848; doi:10.1038/s41467-021-25790-2)
Supplement: Supplementary file 1 — Supplementary Information [file 41467_2021_25790_MOESM1_ESM.pdf]

SUPPLEMENTARY INFORMATION:

Table 1. siRNAs used in this study.

Target gene	Description	Source/Reference/Sequence
ADAR2	SiADAR2-1 (#1)	CCAUUUACUUCUCGAGCAU
ADAR2	SiADAR2-3(#2)	CGCAGAGUUCCUCACUGUA
ADAR2	Si ADAR2-3´ÚTR	AAAGCACAGUCUAUGGAACGCUAAT
CtIP	SiCtIP	GCUAAAACAGGAACGAAUC
SETX	SiSETX	GCCAGAUCGUAUACAAUUA
NT	*Non-target*	Sigma, SHC016
ADAR3	*siADAR3*	CCUUAAAAUAUGAAUUUACAUGUTA
ADAR1	*siADAR1*	CAGAUAAUCAUGACUUAGCAAGAAT

Table 2. Primary antibodies used in this study. WB, western blotting. IF, immunofluorescence. SMART, Single Molecule Analysis of Resection Tracks. IP, immunoprecipitation.

Primary antibody	Supplier	Reference	Application	Concentration
**BrdU (mouse)**	Sigma Aldrich	RPN202	SMART	1:100
**GFP (rabbit)**	Santa Cruz	sc-8334	IF, WB	1:250
**BRCA1 (mouse)**	Santa Cruz	sc-6954	WB, IF, IP	1:500
**CtIP (mouse)**	Santa Cruz	Gift from Richard Baer	IF	1:500
**γH2AX (mouse)**	Cell Signaling	2577L	IF	1:250
**ADAR2 (mouse)**	Santa Cruz	sc-73409	WB, IP	1:500
**RPA2 (mouse)**	Abcam	ab2175	IF	1:500
**53BP1 (rabbit)**	Novus	NB100-304	IF,WB	1:500
**SETX (rabbit)**	Bethyl Laboratories	(A301-105A)	WB, IP	1:1000
**RNAseH1**	Proteintech,	(156061-AP)	WB	1:1000
**α-tubulin (mouse)**	Sigma	T9026	WB	1:50000
**β-actine (rabbit)**	Abcam	Ab8227	WB	1:50000
**S9.6 (D5H6) (mouse)**	Covalab	Mab0105-P	IF/IP	
**Ku80 (mouse)**	Invitrogen	MA1-23314	WB	1:500

Table 3. Secondary antibodies used in this study. WB, western blotting. IF, immunofluorescence. SMART, Single Molecule Analysis of Resection Tracks.

Secondary antibody	Supplier	Reference	Application	Concentration
**Alexa Fluor 594 goat anti-mouse**	Invitrogen	A11032	IF, SMART	1:500
**Alexa Fluor 488 goat anti-rabbit**	Invitrogen	A11034	IF	1:500
**Alexa Fluor 594 goat anti-mouse**	Jackson Immunorese	115-585-003	IF (laser line)	1:100
**Donkey anti-rabbit-FITC**	Jackson Immunoresearch	711-096-152	IF (laser line)	1:100
**IRDye 680RD goat anti-mouse IgG (H+L)**	Li-cor	926-68070	WB	1:5000-1:10000
**AIRDye 800RD goat anti-rabbit IgG (H+L**	Li-cor	926-32211	WB	1:5000-1:10000

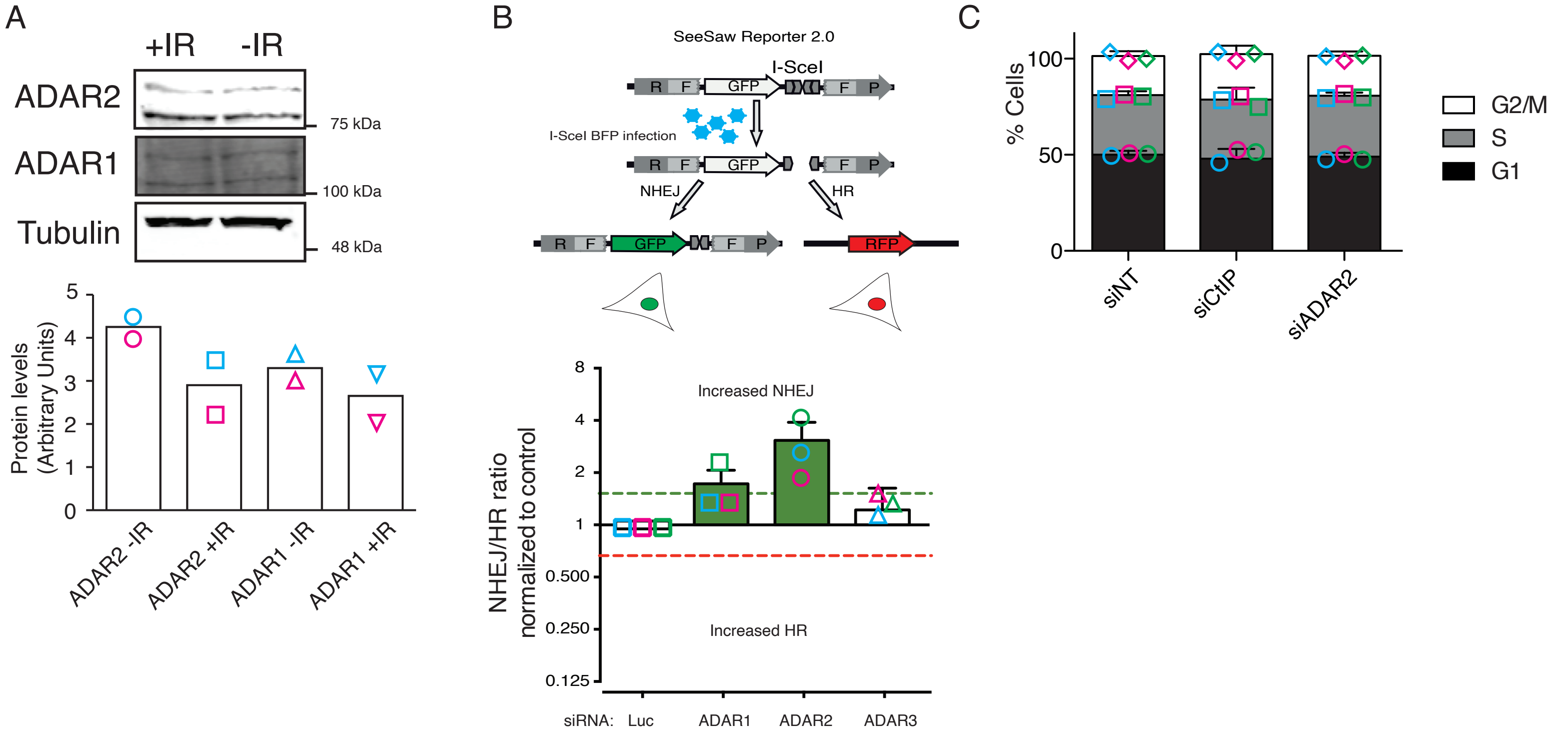

Supplementary Figure 1. ADAR2 unbalance the repair of DSBs. (A) DNA damage does not induce ADAR1 or ADAR2 accumulation. Protein samples from U2OS cells bearing the RNAG reporter collected 1 h after 10 Gy of ionizing radiation (+IR) or mock treated (-IR) were resolved in SDS-PAGE and blotted with antibodies against ADAR1 and ADAR2 or tubulin as a loading control. The plot shows the media of the quantification of protein levels of two different experiments. Each individual replica is marked with a colored symbol. (B) In the top side, a schematic representation of the SeeSaw reporter (SSR). Infection with a virus bearing the I-SceI gene induces the formation of a DSB in the reporter. When repaired by NHEJ, an active GFP gene is reformed. However, the break can be also repaired by intra-strand recombination using two copies of truncated RFP. In that case, the cells will express the RFP protein and lose the GFP gene. On the bottom side, the effect of the depletion of different ADAR family members in the SSR is shown. Data from (1) Each individual replica is marked with a colored symbol. (C) Cell cycle analysis of U2OS cells transfected with the indicated siRNAs measured by FACs. The average and standard deviation of three independent experiments is shown. Each individual replica is marked with a colored symbol.

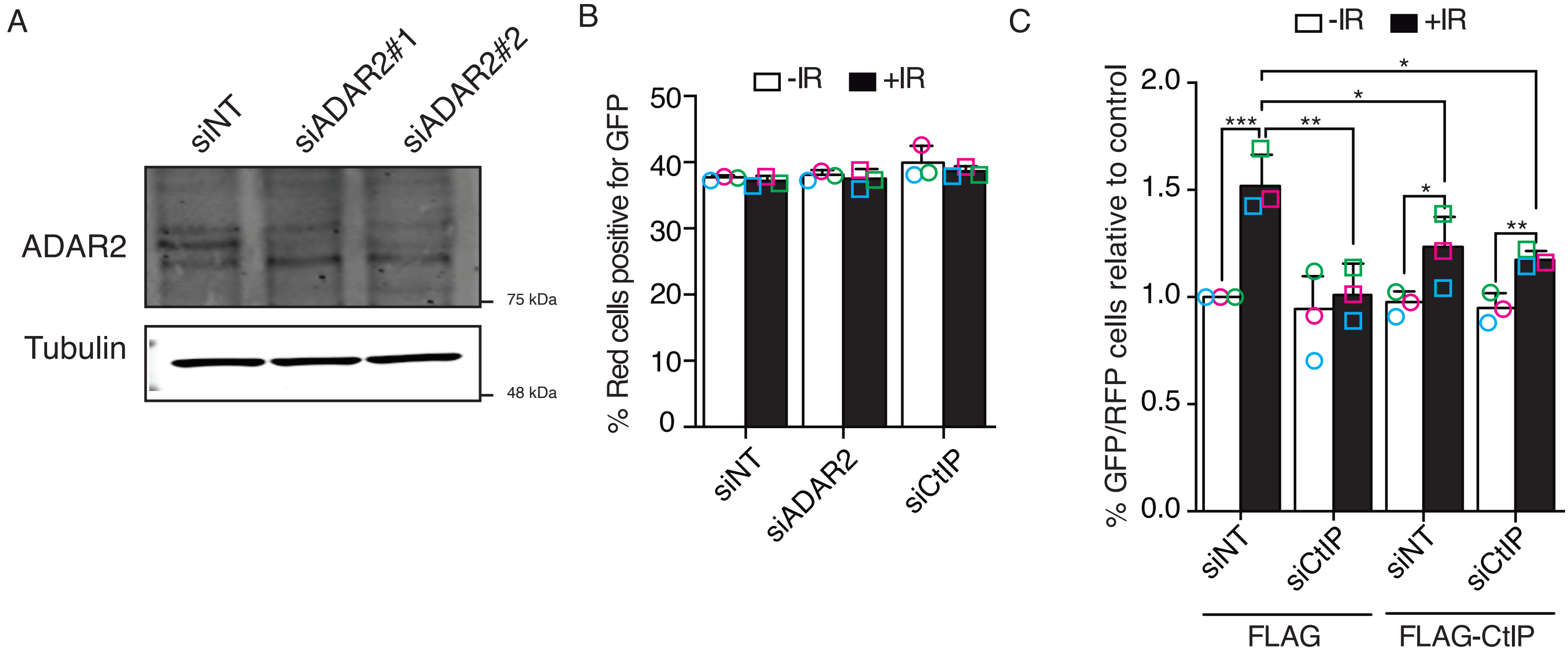

Supplementary Figure 2. DNA damage induced RNA editing. (A) Representative western blot showing siRNA-mediated depletion of ADAR2. U2OS cells were transfected with the indicated siRNAs. 48h later protein samples were obtained, resolved in SDS-PAGE and blotted with the indicated antibodies. The western blot was repeated more than 50 times with identical results. (B) Effect of ADAR2 and CtIP depletion in the control reporter RNWG. Details as in Figure 1D. The average and standard deviation of three independent experiments is shown. Each individual replica is marked with a colored symbol. Lack of statistical significance was determined by two-tailed Student's t-test comparing. (C) Effect of CtIP ectopic expression on DNA damage-induced RNA editing. U2OS bearing the RNAG reporter system were transfected with siCtIP or a control siRNA (siNT) and complemented with a FLAG-CtIP construct or a FLAG empty vector. Other details as in Figure 1. IR: Ionizing Radiation The average and standard deviation of three independent experiments is shown. Each individual replica is marked with a colored symbol.

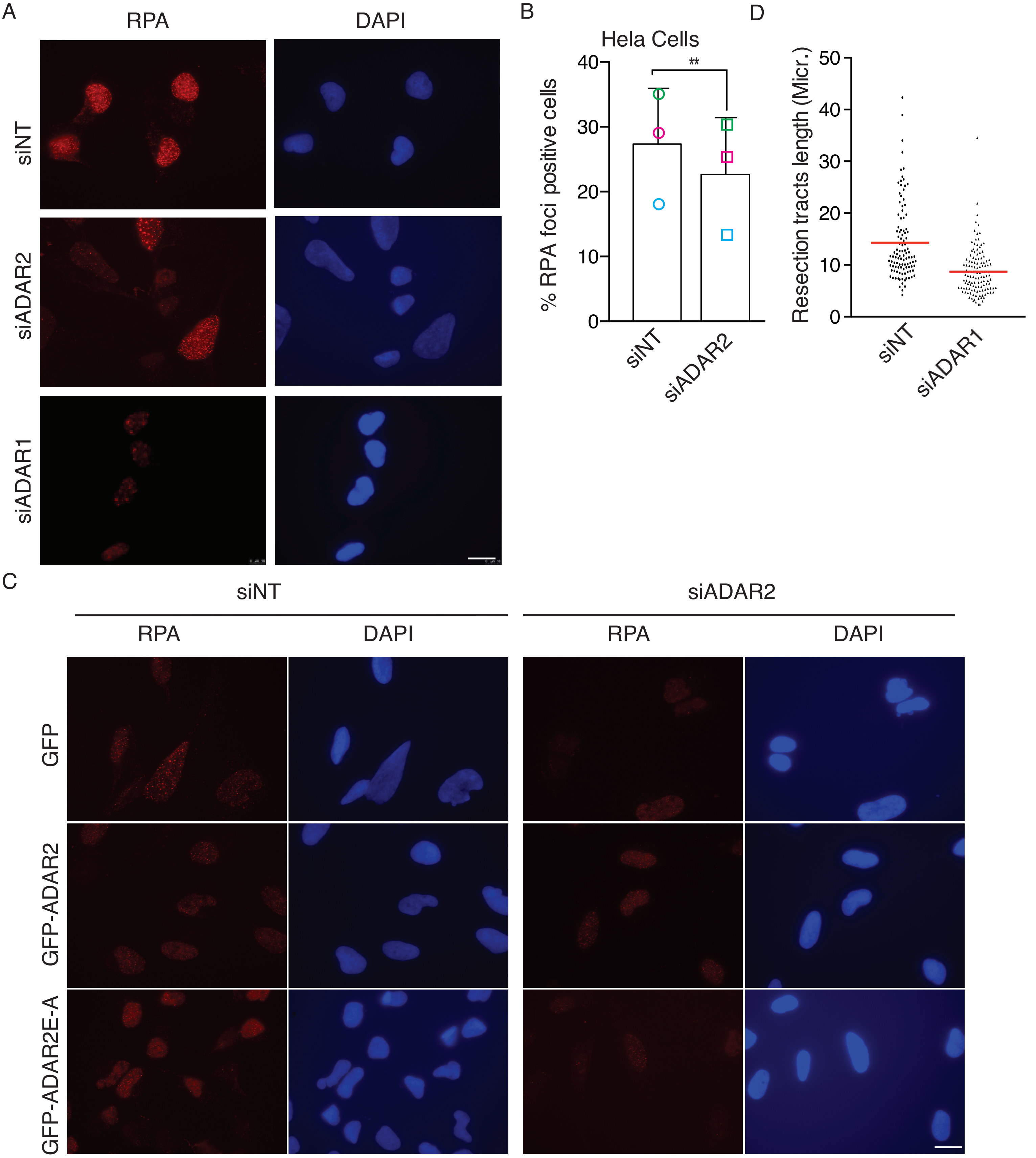

Supplementary Figure 3. ADAR2 depletion impairs DNA end resection. (A) Representative images of the experiment shown in Figure 4A. Scale bars represent 20 μm. (B) RPA foci in HeLA cells depleted for ADAR2. The average and standard deviation of three independent experiments is shown. Each individual replica is marked with a colored symbol. Significance was determined by paired two-tailed Student's t-test comparing each condition to siNT cells. **, $P < 0.01$. Actual p-values can be found in the Source Data File. Other details as in Figure 4A. (C) Representative images of the experiment shown in Figure 4B. Scale bars represent 20 μm. (D) DNA end resection upon downregulation of ADAR1 using SMART. Other details as in figure 4E.

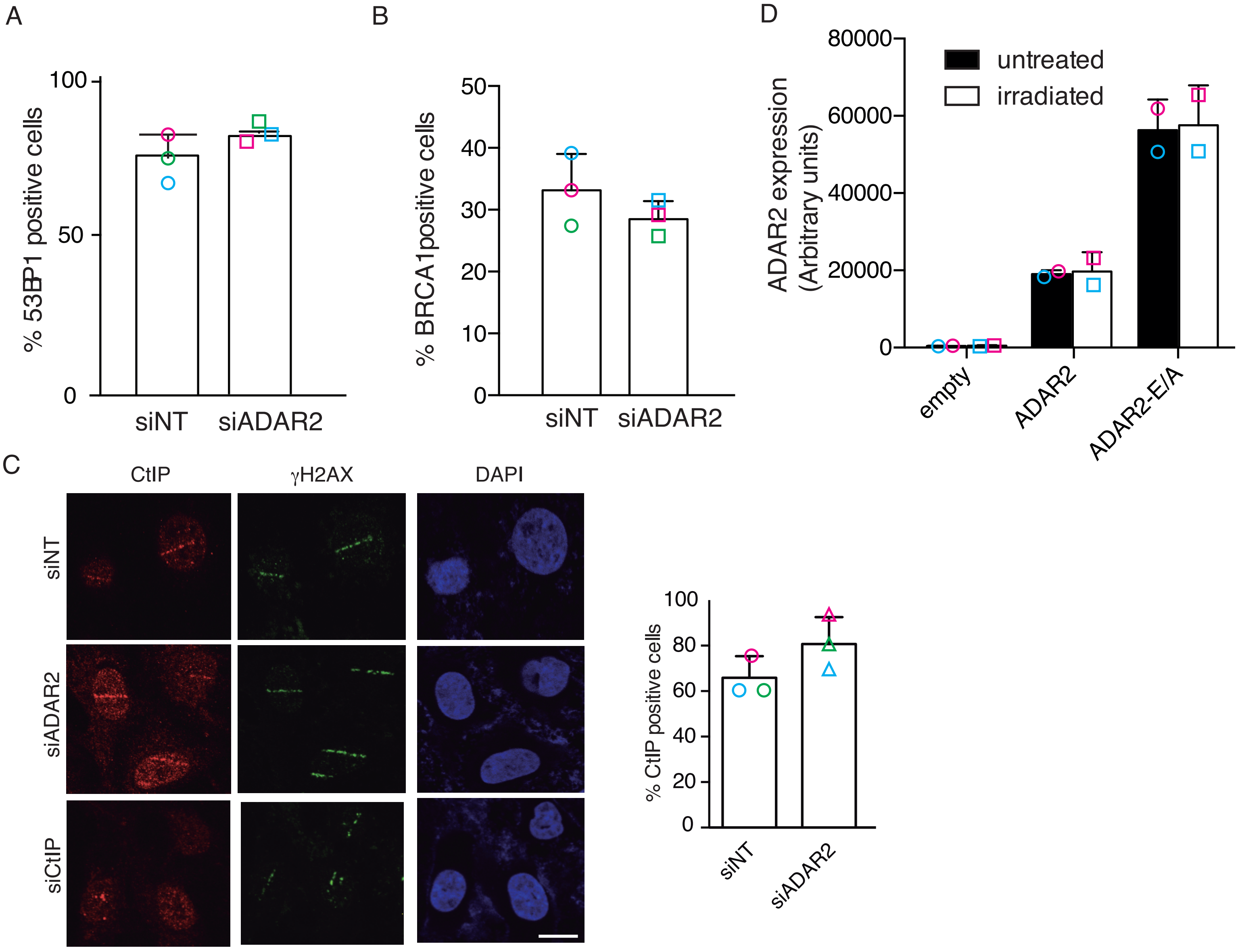

Supplementary Figure 4. ADAR2 depletion does not grossly affects the recruitment of pro- or anti- resection factors. (A) U2OS cells transfected with the indicated siRNAs were irradiated (10 Gy). One hour later, cells were prepared for immunostaining using a 53BP1 antibody. The number of 53BP1 foci-positive cells is plotted. Graph represent the average and standard deviation of three independent experiments. Each individual replica is marked with a colored symbol. (B) Same as A, but using a BRCA1 antibody. The average and standard deviation of three independent experiments is shown. Each individual replica is marked with a colored symbol. (C) Cells were laser-microirradiated as described in the methods section and stained with an antibody against CtIP. Representative images are shown on the left, and the average and standard deviation of three independent experiments is shown on the right. Each individual replica is marked with a colored symbol. Scale bars represent 20 µm. (D) Expression levels of ADAR2 in U118 cells complemented with a plasmid bearing the wildtype gene (ADAR2), a catalytically dead mutant (E/A) or the empty vector. Expression was obtained by RNA sequencing. The average and standard deviation of two independent experiments is shown. Each individual replica is marked with a colored symbol.

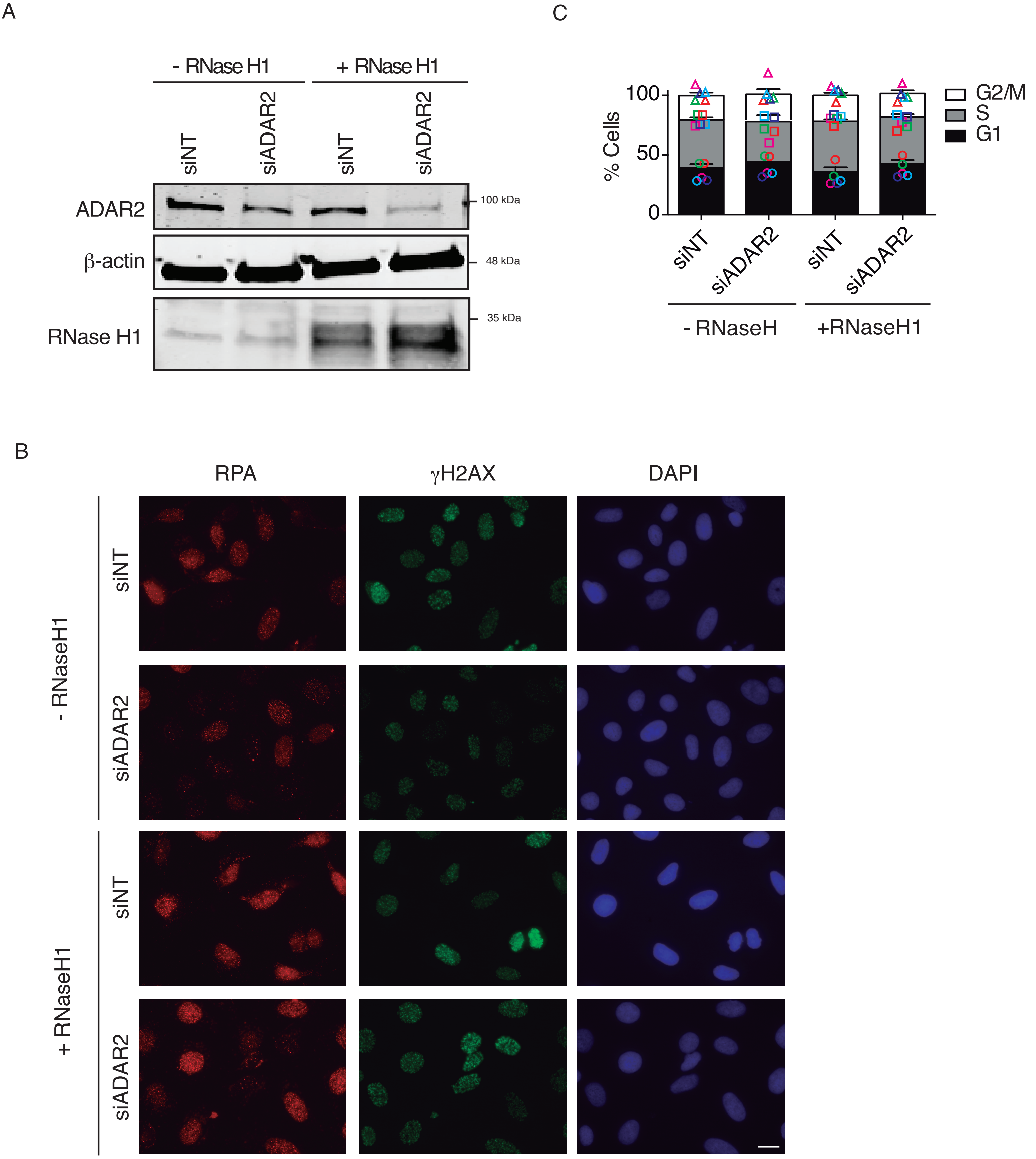

Supplementary Figure 5. ADAR2 resection upon overexpression of RNase H1. (A) Representative western blot of ADAR2 depletion and RNAseH1 overexpression. U2OS cells were transfected with pcDNA3-RNaseH1 or pCDNA3 and depleted or not of ADAR2. Protein samples were isolated, resolved in SDS-PAGE and blotted with the indicated antibodies. A representative western blot out of 12 independent experiments is shown. Scale bars represent 20 µm. (B) Representative images of the experiment shown in Figure 5A. (C) Cell cycle distribution of cells treated as in (A) and measured by FACs analysis. See methods for details. The average and standard deviation of five independent experiments is shown. Each individual replica is marked with a colored symbol.

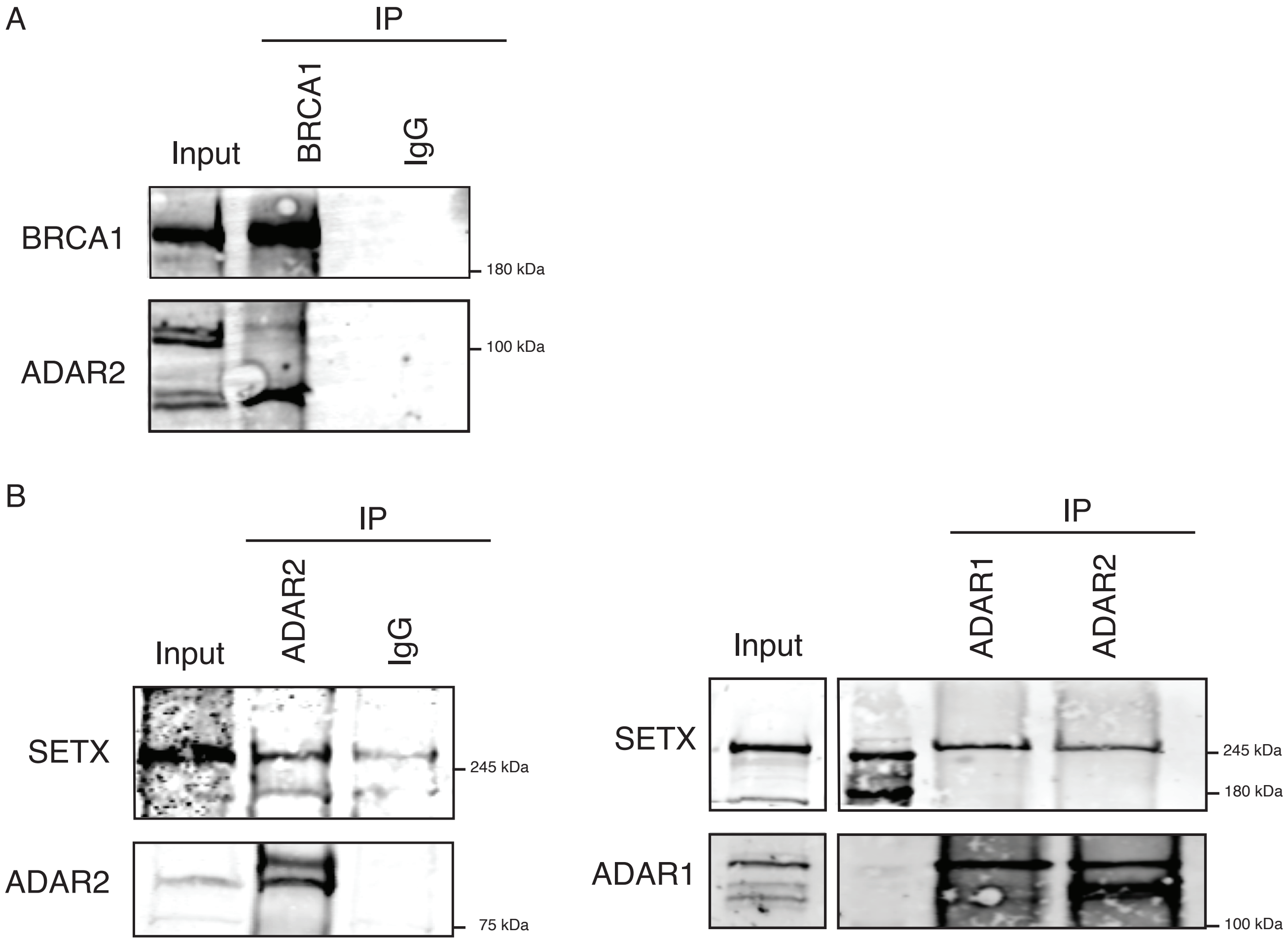

Supplementary Figure 6. Biological replicas of the co-immunoprecipitations showed in figure 6. (A) Protein samples from U2OS cells were immunoprecipitated (IP) using an anti-BRCA1 antibody or a non-related IgG as a control. Input and immunoprecipitates were resolved in SDS-PAGE and blotted for BRCA1 and ADAR2, as indicated. A representative western blot out of three is shown. Source data are provided in the Source Data file. (B) Protein samples from U2OS cells were immunoprecipitated using an anti-ADAR2, an anti-ADAR1 antibody or a non-related IgG as a control, as indicated. Inputs an immunoprecipitates were resolved in SDS-PAGE and blotted for ADAR1, ADAR2 and SETX as marked. Representatives western blots are shown out of three biological replicas. Source data are provided in the Source Data file.

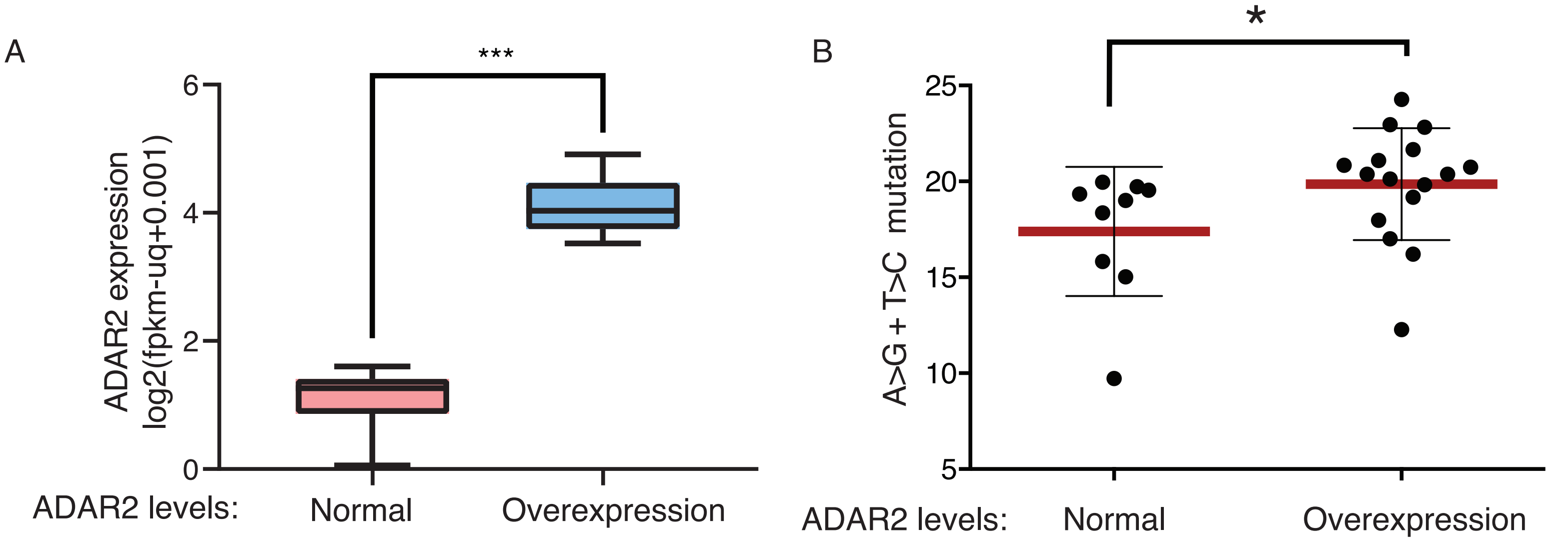

Supplementary Figure 7. Mutagenesis associated to ADAR2 overexpression in cancer cell lines. (A) Stratification of tumor samples regarding ADAR2 expression levels in the control group (ADAR2 WT, n=17) and overexpressing group (ADAR2 +, n=24). Expression levels are expressed as log2(fpkm-uq+0.001). (B) A to G and T to C somatic mutation levels in tumors from the ADAR2 overexpressing (ADAR2 +, n=24) and ADAR2 control group (ADAR2 WT, n=17). A to G and T to C percentage were used as a measurement for ADAR2 impact over the mutational landscape of ADAR2. A to G and T to C percentage was calculated using the total amount of mutations in each donor set. Statistical significance was calculated with a two-tailed Student's t-test with Welsch correction and is denoted with one ($p<0.05$) or three ($p<0.001$) asterisks. Actual p-values can be found in the Source Data file. The average is depicted as a red line and error bars represent the standard deviation.
