# Supplementary material for: ADAR-mediated RNA editing of DNA:RNA hybrids is required for DNA double strand break repair
Source: Nat Commun. 2021 Sep 17;12:5512. doi: 10.1038/s41467-021-25790-2 (PMC8448848; doi:10.1038/s41467-021-25790-2)
Supplement: Supplementary file 2 — Description of Additional Supplementary Files [file 41467_2021_25790_MOESM2_ESM.pdf]

Description of Additional Supplementary Files

File Name: Supplementary Data 1
Description: Recoding index changes upon irradiation. A list of all genes which recoding index significantly changes upon irradiation, with the percentage of recoding in non-irradiated samples and irradiated samples.
