# Supplementary material for: ADAR-mediated RNA editing of DNA:RNA hybrids is required for DNA double strand break repair
Source: Nat Commun. 2021 Sep 17;12:5512. doi: 10.1038/s41467-021-25790-2 (PMC8448848; doi:10.1038/s41467-021-25790-2)
Supplement: Supplementary file 4 — Reporting Summary [file 41467_2021_25790_MOESM4_ESM.pdf]

nature research

Corresponding author(s): Pablo Huertas

Last updated by author(s): 10/03/2021

Reporting Summary

Nature Research wishes to improve the reproducibility of the work that we publish. This form provides structure for consistency and transparency in reporting. For further information on Nature Research policies, see our Editorial Policies and the Editorial Policy Checklist.

Statistics

For all statistical analyses, confirm that the following items are present in the figure legend, table legend, main text, or Methods section.

n/a	Confirmed	
☐	☒	The exact sample size (n) for each experimental group/condition, given as a discrete number and unit of measurement
☐	☒	A statement on whether measurements were taken from distinct samples or whether the same sample was measured repeatedly
☐	☒	The statistical test(s) used AND whether they are one- or two-sided<br>*Only common tests should be described solely by name; describe more complex techniques in the Methods section.*
☒	☐	A description of all covariates tested
☒	☐	A description of any assumptions or corrections, such as tests of normality and adjustment for multiple comparisons
☐	☒	A full description of the statistical parameters including central tendency (e.g. means) or other basic estimates (e.g. regression coefficient) AND variation (e.g. standard deviation) or associated estimates of uncertainty (e.g. confidence intervals)
☐	☒	For null hypothesis testing, the test statistic (e.g. F, t, r) with confidence intervals, effect sizes, degrees of freedom and P value noted<br>*Give P values as exact values whenever suitable.*
☒	☐	For Bayesian analysis, information on the choice of priors and Markov chain Monte Carlo settings
☒	☐	For hierarchical and complex designs, identification of the appropriate level for tests and full reporting of outcomes
☒	☐	Estimates of effect sizes (e.g. Cohen's d, Pearson's r), indicating how they were calculated

Our web collection on statistics for biologists contains articles on many of the points above.

Software and code

Policy information about availability of computer code

Data collection	Mutations sets were retrieved from the International Cancer Genome Consortium (ICGC) Data Portal MALY-DE and CLLE-ES datasets. ADAR2 expression levels from each donor were obtained using the UCSC Xena web tool.
Data analysis	Open-access somatic mutations information from each mutation set was obtained by comparing each mutation set with the latest release of the Aggregated Somatic Mutation VCF file by the ICGC using custom python scripts.<br>RNA editing analysis was performed using a specific python tool, REDItools

For manuscripts utilizing custom algorithms or software that are central to the research but not yet described in published literature, software must be made available to editors and reviewers. We strongly encourage code deposition in a community repository (e.g. GitHub). See the Nature Research guidelines for submitting code & software for further information.

Data

Policy information about availability of data

All manuscripts must include a data availability statement. This statement should provide the following information, where applicable:

- Accession codes, unique identifiers, or web links for publicly available datasets
- A list of figures that have associated raw data
- A description of any restrictions on data availability

All relevant data are included in the manuscript. Raw data will be provided upon request.

Field-specific reporting

Please select the one below that is the best fit for your research. If you are not sure, read the appropriate sections before making your selection.

☒ Life sciences ☐ Behavioural & social sciences ☐ Ecological, evolutionary & environmental sciences

For a reference copy of the document with all sections, see nature.com/documents/nr-reporting-summary-flat.pdf

Life sciences study design

All studies must disclose on these points even when the disclosure is negative.

Sample size	No sample size calculation was performed
Data exclusions	No data were excluded
Replication	All replicas showed similar results and all the data derived from them have been included in the analysis.
Randomization	This is was not required for our study
Blinding	No blinding was performed, as it is not required for our studies.

Reporting for specific materials, systems and methods

We require information from authors about some types of materials, experimental systems and methods used in many studies. Here, indicate whether each material, system or method listed is relevant to your study. If you are not sure if a list item applies to your research, read the appropriate section before selecting a response.

Materials & experimental systems

n/a	Involved in the study	
☐	☒	Antibodies
☐	☒	Eukaryotic cell lines
☒	☐	Palaeontology and archaeology
☒	☐	Animals and other organisms
☒	☐	Human research participants
☒	☐	Clinical data
☒	☐	Dual use research of concern

Methods

n/a	Involved in the study	
☒	☐	ChIP-seq
☐	☒	Flow cytometry
☒	☐	MRI-based neuroimaging

Antibodies

Antibodies used	All this information can be found in the supplemental material.
Validation	All antibodies were previously validated by using downregulated cells as controls

Eukaryotic cell lines

Policy information about cell lines

Cell line source(s)	All cell lines were obtained from ACGT, with the exception of U118 and derivated cell lines, that were obtained and validated previously by one of the authors, A. Gallo.
Authentication	No cell lines were further authenticated.
Mycoplasma contamination	We test for mycoplasma infection routinely in the lab.
Commonly misidentified lines (See ICLAC register)	None

Flow Cytometry

Plots

Confirm that:

☒ The axis labels state the marker and fluorochrome used (e.g. CD4-FITC).

☒ The axis scales are clearly visible. Include numbers along axes only for bottom left plot of group (a 'group' is an analysis of identical markers).

☐ All plots are contour plots with outliers or pseudocolor plots.

☐ A numerical value for number of cells or percentage (with statistics) is provided.

Methodology

Sample preparation	All is detailed in the methods section
Instrument	FACSCalibur (BD). ; FACSAriaTM
Software	BD FACSDiva Software<br>ModFit LT 3.0 software
Cell population abundance	No post-sort was performed
Gating strategy	None

☐ Tick this box to confirm that a figure exemplifying the gating strategy is provided in the Supplementary Information.
